# Chronic ethanol consumption and HBV induce abnormal lipid metabolism through HBx/SWELL1/arachidonic acid signaling and activate Tregs in HBV-Tg mice

**DOI:** 10.7150/thno.46005

**Published:** 2020-07-23

**Authors:** Zixian Liu, Jiapei Wang, Lei Liu, Hongfeng Yuan, Yanan Bu, Jinyan Feng, Yunxia Liu, Guang Yang, Man Zhao, Ying Yuan, Huihui Zhang, Haolin Yun, Xiaodong Zhang

**Affiliations:** Department of Cancer Research, College of Life Sciences, Nankai University, Tianjin 300071, P.R. China.

**Keywords:** Ethanol, HBx, SWELL1, Arachidonic acid metabolism, Tregs

## Abstract

**Rationale:** Chronic ethanol consumption as a public health problem worldwide boosts the development of chronic liver diseases in hepatitis B virus (HBV)-infected patients. Arachidonic acid metabolite prostaglandin E2 (PGE2) activates regulatory T cells (Tregs) function. Here, we aim to investigate the underlying mechanism by which chronic ethanol consumption enriches the HBV-induced abnormal lipid metabolism and Tregs.

**Methods:** The si-RNAs were used to weaken the expression of SWELL1 in HepG2, HepG2.2.15 and K180 cancer cell lines, followed by RNA sequencing from HepG2 cells. Arachidonic acid metabolite PGE2 and LTD4 were measured by ELISA assay *in vivo* and *in vitro.* Western blot analysis and RT-qPCR were used to examine HBx and SWELL1 and transcriptional factor Sp1 in clinical HCC samples and cell lines. The effect of chronic ethanol consumption on Tregs was tested by flow cytometry in HBV-Tg mice. The splenic Tregs were collected and analyzed by RNA sequencing.

**Results:** The cooperative effect of ethanol and HBV in abnormal lipid metabolism was observed* in vivo* and *in vitro*. The depression of SWELL1 (or HBx) resulted in the reduction of lipid content and arachidonic acid metabolite, correlating with suppression of relative gene atlas. Ethanol and SWELL1 elevated the levels of PGE2 or LTD4 in the liver of mice and cell lines. Interestingly, the ethanol modulated abnormal lipid metabolism through activating HBx/Sp1/SWELL1/arachidonic acid signaling. Chronic ethanol consumption remarkably increased the population of PBL Tregs and splenic Tregs in HBV-Tg mice, consistently with the enhanced expression of PD-L1 *in vivo* and *in vitro*. Mechanically, RNA-seq data showed that multiple genes were altered in the transcriptomic atlas of Tregs sorting from ethanol-fed mice or HBV-Tg mice.

**Conclusion:** The chronic ethanol intake enriches the HBV-enhanced abnormal lipid metabolism through HBx/SWELL1/arachidonic acid signaling and activates Tregs in mice.

## Introduction

Chronic ethanol consumption and hepatitis B virus (HBV) infection, both recognized as the main causes of chronic liver disease, frequently coexist in the patients with chronic liver diseases 1. Alcoholic liver disease (ALD) is a major global burden and the spectrum of ALD includes alcoholic fatty liver disease, alcoholic hepatitis, alcohol related liver fibrosis and cirrhosis 2. Both of ethanol intake and HBV target the liver and may result in hepatitis, cirrhosis, and hepatocellular carcinoma (HCC) at last. Accordingly, there is a 2- to 4-fold higher frequency of HBV markers in the serum of chronic alcoholics, and among patients with alcoholic hepatitis 3 and cirrhosis 4, compared with the general population.

Chronic ethanol consumption frequently displays metabolic reprogramming and acquires constitutively sustained nutrition uptake 5. Lipids, functioning as essential structural components of membranes and serving as important energy resources, are critical macromolecules for cell proliferation 6. The finding that HBV-infected alcoholics develop chronic liver disease (CLD) and HCC at an earlier age than uninfected alcoholics reflects the synergy between HBV and ethanol in liver cell damage and disease progression 5, 7. HBV expression synergistically increased cholesterol deposition in the setting of alcoholic fatty liver. Additionally, HBV infection aggravates ethanol-induced cytotoxicity in habitual drinkers. However, the underlying mechanism of the interaction of ethanol and HBV is still obscure 8, 9. Moreover, sporadic lines of evidence demonstrate that the chronic ethanol consumption disrupts cholesterol homeostasis 10. Abnormal metabolism widely influences the development of liver damage. Although HBV has been described as a typical non-cytopathic virus, it can induce tissue damage of variable severity by stimulating a protective immune response, that can simultaneously cause damage and protection 11.

Elevated lipid biosynthesis promotes the expression of effector molecules and the immunosuppressive activity of regulatory T cells (Tregs), which enables them to control conventional T cells to maintain immune homeostasis 12. As a production of arachidonic acid matabolism, prostaglandin E2 (PGE2) promotes Treg activities in human lymphocytes, contributing to the tumor-induced immunosuppression 13-15. Excessive ethanol consumption and HBV intracellular persistence synergistically contribute to the synthesis increase, intake and degradation decrease for cholesterol 10. Currently, it lacks large scale epidemiological investigation of these comorbidities, furthermore, the study on the interplay between metabolism and Tregs and consequently the underlying mechanism by which the abnormal lipid metabolism drives immune response is limited.

Tregs, crucial for peripheral tolerance, are intimately involved in the immunological diseases and liver diseases which exploit multiple immunosuppressive pathways to actively evade immune recognition, including endogenous “immune checkpoints” that normally terminate immune responses after antigen activation 16, 17. Programmed death 1 (PD-1) is an inhibitory checkpoint receptor, for that the engagement of PD-1 on T cells with programmed death ligand 1 (PD-L1) on tumor cells down-regulates antitumor T cell responses. The enhanced Treg activity is usually associated with poor immune responses to antigens, and the up-regulation of PD-L1 by neoplastic cells allows tumors to escape the antitumor effector T cell responses 18. Tregs can contribute to sustain a state of virus-specific T cell collapse, which is a characteristic of chronic hepatitis B (CHB) infection. Therefore, the presence of HBV-specific Treg population can represent a hallmark for inappropriate adaptive immune response during CHB infection 19. However, the underlying mechanism is poorly understood. Therefore, the identification of the potencial regulators, systematically detecting the synergistically effects of chronic ethanol consumption and HBV in lipid metabolic disorder and concequently Treg response, is needed* in vivo*.

The HBV genome is composed of four open reading frames 20, 21. Among them, hepatitis B virus X protein (HBx), as a multifunctional regulation factor, plays crucial roles in the viral pathogenesis and carcinogenesis of HCC 21-23. Our laboratory reported that HBx accelerated the lipogenesis in hepatoma cells 24-26. However, the underlying mechanism of liver damage caused by ethanol and HBV is elusive.

As one of the components forming volume-regulated anion channel, SWELL1 (also named the leucine-rich repeat-containing protein 8A, LRRC8A) plays a crucial role in T cell development, pancreatic B-cell glucose-stimulated insulin secretion, and adipocyte metabolic function 27. Interestingly, recent study shows that the volume-sensitive SWELL1 participates in sensing adipocyte volume during physiological or pathophysiological adipocyte expansion and engages insulin-PI3K-AKT signaling, thereby coupling adipocyte growth with insulin signaling, glucose uptake and lipid content 28. Notwithstanding the key role of SWELL1 expression by adipocyte cells, our understanding of the regulation of SWELL1 in modulation of lipogenesis in hepatoma cells is unclear.

In this study, we are interested in the combination effect of chronic ethanol consumption with HBV on abnormal lipid metabolism and Tregs using a model of HBV transgenic (HBV-Tg) mice. Strikingly, we found that the ethanol modulated the abnormal lipid metabolism through activating HBx/Sp1/SWELL1/arachidonic acid signaling in liver. Meanwhile, the chronic ethanol consumption was able to increase the HBV-induced Treg population in the system. Our finding provides new insights into the mechanism by which chronic ethanol consumption and HBV enrich abnormal lipid metabolism and activate Tregs in mice.

## Results

### Ethanol enriches HBV-enhanced abnormal lipid metabolism involving HBx and SWELL1

**Figure 1:**
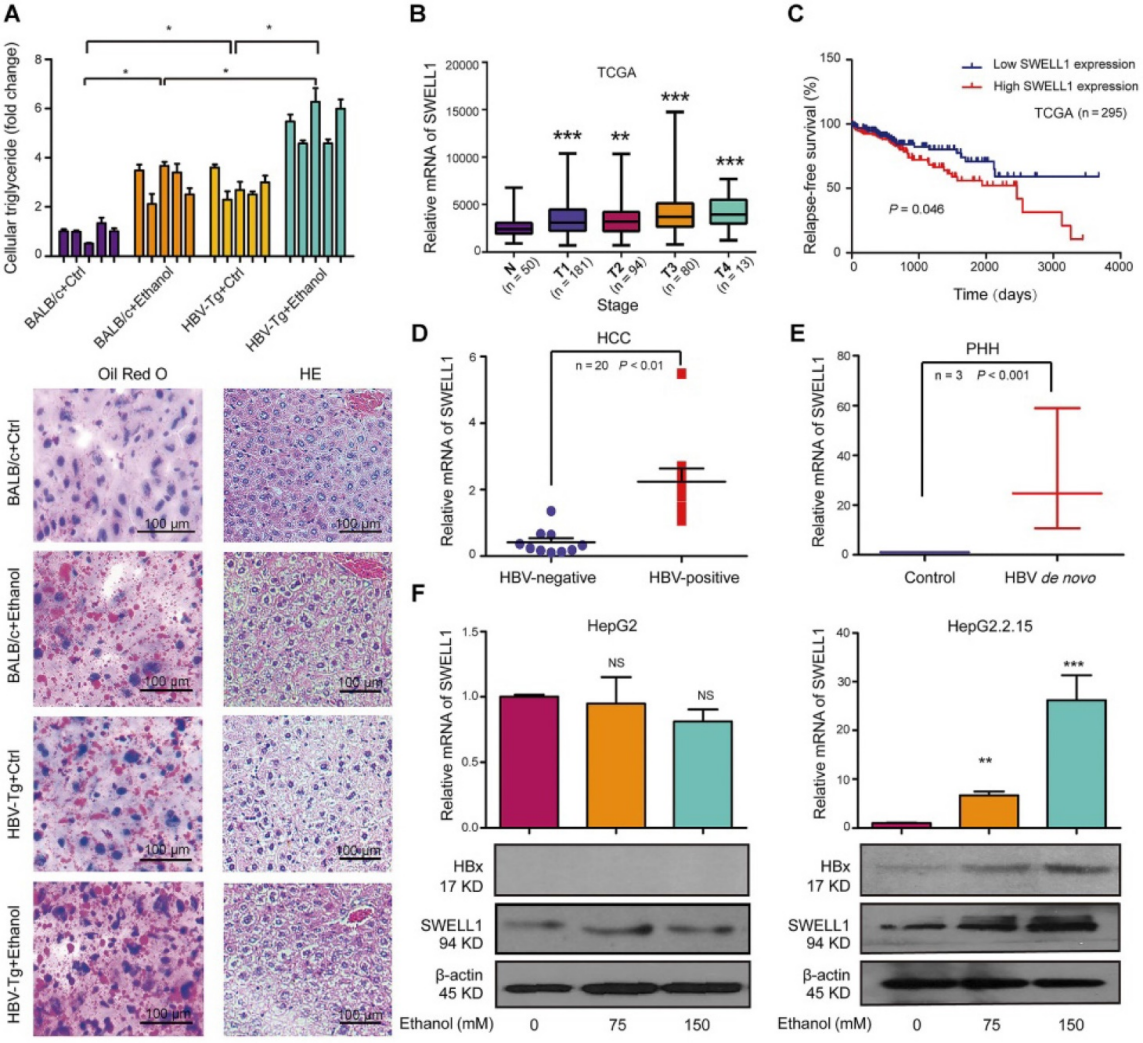
** Ethanol enriches the HBV-enhanced abnormal lipid metabolism involving HBx and SWELL1.** (**A**) Top panel: The levels of triglycerides were individually measured in the liver tissues from four groups of mice (n = 5). Bottom panel: Lipogenesis in the liver tissues from mice was determined by Oil Red O staining and tissue pathology of the liver was evaluated by HE staining using frozen sections. (**B**) DRG analysis of TCGA HCC patients with SWELL1 expression. The number of HCC patients in each stage was labeled, respectively. (**C**) Relapse-free survival analysis of TCGA HCC patients with low versus high SWELL1 expression (n = 295, *P* = 0.046). (**D**) The mRNA levels of SWELL1 in HBV-negative HCC tissues or HBV-positive HCC tissues were assessed by RT-qPCR (n = 20, *P* < 0.01). (**E**) The mRNA levels of SWELL1 were assessed by RT-qPCR in PHH cells with *de novo* HBV infection and the normal PHH cells were used as control. (**F**) The mRNA and protein levels of SWELL1 were determined by RT-qPCR and Western blot analysis in HepG2 cells or HepG2.2.15 cells dose-dependently treated with ethanol. Each experiment was repeated at least three times. Error bars represent means ± SD (n = 3). *P < 0.05; **P < 0.01; ***P < 0.001; NS, no significant; Student's *t*-test.

In this study, we were interested in the role of chronic ethanol consumption in modulation of HBV-enhanced abnormal metabolism in liver. Basically, lipid droplets can be used as a marker for lipogenesis. Liver pathology using hematoxylin-eosin (HE) staining and Oil Red O staining evinced that the lipid droplets were increased in the liver tissues from pair-fed HBV-Tg mice and chronically ethanol feeding BALB/c mice, especially in the chronically ethanol feeding HBV-Tg mice (**Figure 1A**, bottom panel). Lipid droplets form the main lipid store including triglyceride and cholesterol in eukaryotic cells 29. Our data showed that the chronic ethanol consumption alone induced an increase of triglyceride level and cholesterol level in the liver from mice, compared with the pair-fed control mice. And chronic ethanol consumption combined with HBV further significantly elevated the above lipid content in the liver tissues, relative to the other three groups (**Figure 1A and Figure S1A**). HBV DNA integration was frequently detected in clinical samples, therefore, HBV DNA integrated HepG2.2.15 cell line was used in this study 30, 31. We aimed to explore the role of alcohol consumption in stimulating abnormal lipid metabolism in the context of HBV integration. Our finding showed that the treatment with ethanol enhanced the levels of lipid droplets in HepG2 cells and HepG2.2.15 cells in a dose-dependent manner. Obviously, the ethanol caused a higher level of accumulation of lipid droplets in HBV-expressing hepatoma cells than that in HepG2 cells (**Figure S1B**). We further evaluated the effect of ethanol on the cellular triglyceride or cellular cholesterol in HepG2 cells and HepG2.2.15 cells. The similar results of cellular triglyceride and cholesterol were observed in the system (**Figure S1C-D**), suggesting that the ethanol and HBV cooperatively increase the abnormal lipid metabolism in HBV-Tg mice and in the cell lines.

Next, we supposed that SWELL1 might play a crucial role in modulation of abnormal lipid metabolism in our system, because SWELL1 modulated lipid content in normal adipocyte cells 28. The analysis of a publicly available HCC patient data from The Cancer Genome Atlas (TCGA) in liver cancers revealed that SWELL1 was an important factor of evaluating risk of mortality and Diagnosis Related Grading (DRG) of HCC patients, in which the up-regulation of SWELL1 was observed in every stage of HCC patients (**Figure 1B**). We further analyzed the HCC patient TCGA dataset as previously described 32. Strikingly, the high expression of SWELL1 was significantly correlated with a poor relapse-free survival (*P* = 0.046, **Figure 1C**). Clinically, we showed that the expression levels of SWELL1 were higher in HBV-positive HCC samples than those in HBV-negative HCC ones (**Figure 1D**). Interestingly, we demonstrated that the expression levels of SWELL1 were significantly elevated in primary human hepatocytes (PHH) infected with HBV (**Figure 1E**), suggesting that HBV may up-regulate SWELL1 in liver. Meanwhile, ELISA assays indicated that the levels of HBeAg and HBsAg were significantly increased in PHH cells infected with HBV, to ensure the HBV infection efficiency (**Figure S1E-F**). And the levels of HBx/pgRNA were dose-dependently elevated by ethanol treatment in HBV-expressing HepG2.2.15 cells (**Figure S1G**). As one of the HBV viral proteins, HBx plays crucial roles during the abnormal lipid metabolism development of HCC. We further evaluated the effect of ethanol on HBx or SWELL1. Interestingly, our data showed that the treatment with ethanol was able to up-regulate HBx and SWELL1 at the levels of mRNA and protein in HBV-expressing HepG2.2.15 cells in a dose-dependent manner, but not in HBV-free HepG2 cells (**Figure 1F**), suggesting that HBx and SWELL1 are involved in the ethanol-induced event. Thus, we conclude that the ethanol increases the levels of HBx and SWELL1 in HBV-expressing hepatoma cells and HBV increases the levels of SWELL1 in PHH.

### HBx up-regulates SWELL1 through co-activating transcription factor Sp1

**Figure 2:**
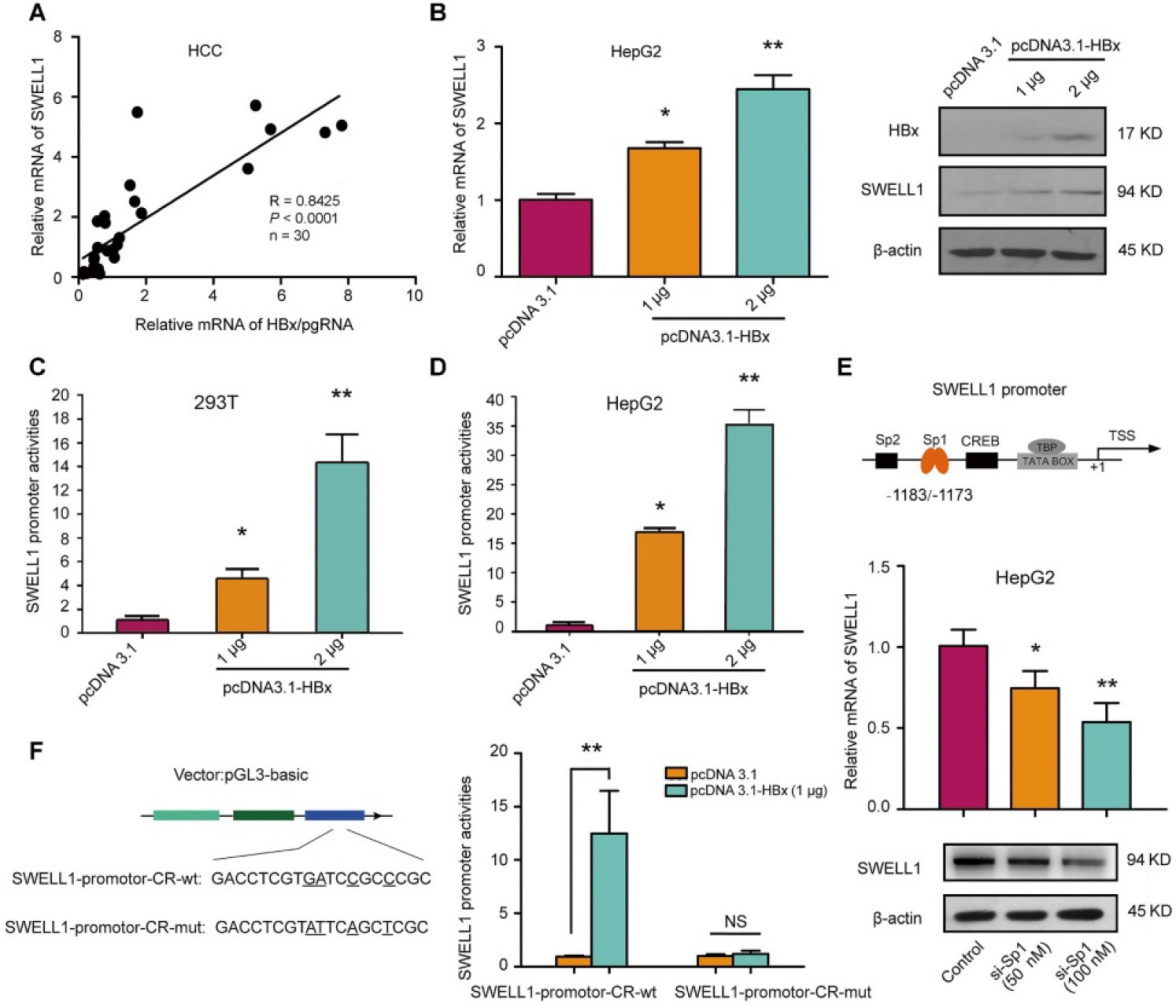
** HBx up-regulates SWELL1 through co-activating transcription factor Sp1.** (**A**) Correlation of mRNA levels between HBx/pgRNA and SWELL1 was examined by RT-qPCR in HCC clinical tissues (n = 30, R = 0.8425, *P* < 0.0001, Pearson's correlation coefficient). (**B**) The mRNA and protein levels of SWELL1 were assessed by RT-qPCR and Western blot analysis in HepG2 cells, respectively. (**C and D**) Luciferase reporter gene assays were performed in 293T cells or HepG2 cells transiently transfected with pGL3-SWELL1-promoter (0.2 µg/well) and treated with indicated concentrations of pcDNA3.1-HBx. (**E**) Top panel: A model of transcription factor locus represents putative target sites for SWELL1 promoter. Bottom panel: The mRNA and protein levels of SWELL1 were assessed by RT-qPCR and Western blot analysis in HepG2 cells transfected with siRNA of Sp1 in dose-dependent treatment (0, 50 nM, 100 nM), respectively. (**F**) Left panel: A model of molecular constitution represents SWELL1-promoter-CR-wt and SWELL1-promoter-CR-mut. Right panel: Luciferase reporter gene assays were used to measure the promoter activity of SWELL1 in HepG2 cells transiently transfected with pGL3-SWELL1-promoter-CR-wt or pGL3-SWELL1-promoter-CR-mut (0.2 µg/well) and treated with indicated concentrations of pcDNA3.1-HBx. Each experiment was repeated at least three times. Error bars represent means ± SD (n = 3). *P < 0.05; **P < 0.01; NS, no significant; Student's *t*-test.

Clinically, our data showed that the expression levels of SWELL1 mRNA were positively associated with those of HBx/pgRNA in 30 clinical HCC tissues (**Figure 2A**). Moreover, the overexpression of HBx dose-dependently resulted in the up-regulation of SWELL1 at the levels of mRNA and protein in HepG2 cells or HepG2.2.15 cells (**Figure 2B and Figure S2A**). Furthermore, the overexpression of HBx resulted in the activation of SWELL1 promoter in 293T cells or HepG2 cells in a dose-dependent manner (**Figure 2C-D**), suggesting that HBx is able to up-regulate SWELL1 in the cells. Based on bioinformatics analysis 33, we obtained several transcriptional factors which potentially bound to the promoter region of SWELL1 from TargetScan (http://www.targetscan.org/) (**Figure 2E**, top panel). Sp1 participates in regulating the expression of genes associated with a wide range of cellular processes in mammalian cells, as a basal transcription factor 34. Previously, our laboratory reported that HBx was able to up-regulate Lin28A/Lin28B through activation of Sp1 35. Therefore, we speculated that Sp1 might be involved in the event that HBx modulated SWELL1. As expected, the mRNA and protein levels of SWELL1 were down-regulated by Sp1 small interfering RNA (siRNA) in a dose-dependent manner (50 nM or 100 nM) in HepG2 cells (**Figure 2E**, bottom panel).

Then, we constructed five fragments binding Sp1 sites in the SWELL1 promoter, respectively (**Figure S2B**). Luciferase reporter gene assays exhibited that Sp1 could activate SWELL1 promoter (position -1381/+215) and the fragment of -1381/-781, more than the other three promoter regions (position -781/+215, position -600/+215 and -200/+215), suggesting that the fragment of -1381/-781 is the core region of SWELL1 promoter. Then, we found that Sp1 binding site located in the SWELL1 upstream (-1183/-1173 nt) was conserved and could be detected by above database. Then, we presumed that HBx might co-activate Sp1 to stimulate SWELL1 promoter. Next, we cloned the core region into the pGL3-basic vector (termed SWELL1-promoter-CR-wt), and validated the effect of HBx overexpression on SWELL1 promoter activity. However, HBx failed to work when the Sp1-binding site in SWELL1 promoter core region was disrupted (termed SWELL1-promoter-CR-mut) (**Figure 2F**). Functionally, our data showed that Sp1 siRNA was able to significantly decrease the levels of cellular triglyceride and cholesterol in HepG2 cells in a dose-dependent manner (**Figure S2C-D**). Therefore, we conclude that HBx activates SWELL1 through co-stimulating transcriptional factor Sp1 in the cells.

### Ethanol enhances abnormal lipid metabolism through HBx/SWELL1 signaling

**Figure 3:**
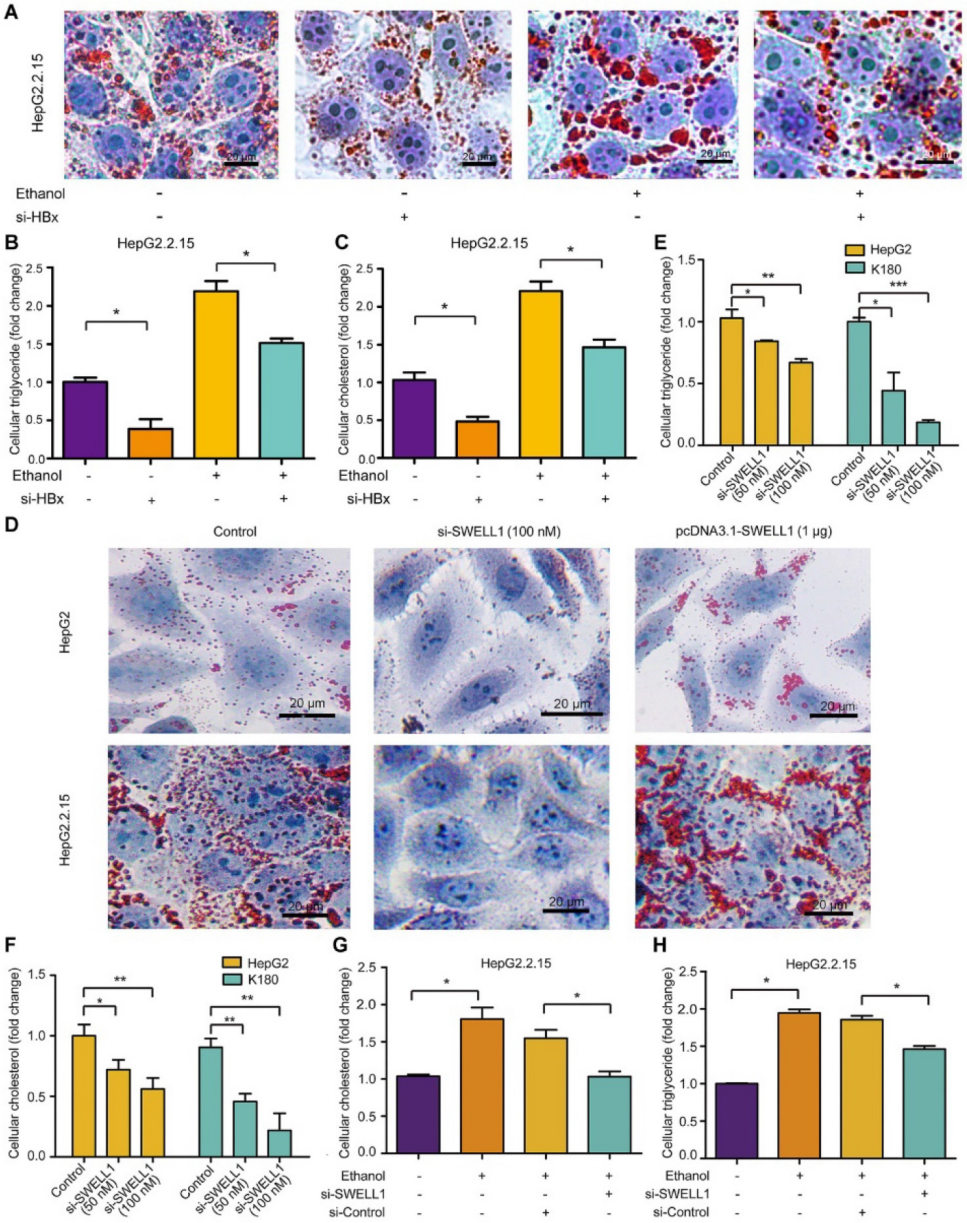
** Ethanol enhances abnormal lipid metabolism through HBx/SWELL1 signaling.** (**A**) Oil Red O staining was used to examine the effect of HBx siRNA, ethanol or both on liquid droplets in HepG2.2.15 cells. (**B**) The effect of HBx siRNA, ethanol or both on cellular triglyceride was measured by ELISA in HepG2.2.15 cells, respectively. (**C**) The effect of HBx siRNA, ethanol or both on cellular cholesterol was detected by ELISA in HepG2.2.15 cells, respectively. (**D**) Oil Red O staining was used to examine the effect of SWELL1 siRNA or pcDNA3.1-SWELL1 on liquid droplets in HepG2 cells and HepG2.2.15 cells. (**E**) The effect of SWELL1 siRNA on cellular triglyceride was measured by ELISA in HepG2 cells and K180 cells. (**F**) The effect of SWELL1 siRNA on cellular cholesterol was measured by ELISA in HepG2 cells and K180 cells. (**G**) The effect of ethanol, SWELL1 siRNA or both on cellular cholesterol was measured by ELISA in HepG2.2.15 cells, respectively. (**H**) The effect of ethanol, SWELL1 siRNA or both on cellular triglyceride was detected by ELISA in HepG2.2.15 cells, respectively. Each experiment was repeated at least three times. Error bars represent means ± SD (n = 3). *P < 0.05; **P < 0.01; ***P < 0.001; Student's *t*-test.

Next, we investigated the underlying mechanism by which ethanol modulated abnormal lipid metabolism in the cells. Interestingly, we showed that knocking down of HBx by si-HBx could significantly block the ethanol-mediated lipid droplet accumulation and levels of triglyceride and cholesterol in HepG2.2.15 cells (**Figure 3A-C**), suggesting that the chronic ethanol consumption with HBV induces abnormal lipid metabolism through HBx. Moreover, Oil Red O assays showed that the treatment with SWELL1 siRNA could efficiently block the lipogenesis in HepG2 cells and HepG2.2.15 cells. Inversely, the overexpression of SWELL1 by pcDNA3.1-SWELL1 was able to heighten the event in the cells (**Figure 3D**). Considering that the specific and potent chemical antagonists and agonists are still not available 36, SWELL1 siRNA was able to significantly reduce the levels of cellular triglyceride and cholesterol in HepG2 cells, HepG2.2.15 cells and esophageal cancer cell line K180 (**Figure 3E-F and Figure S3A**), implying that SWELL1 may broadly lead to abnormal lipid metabolism in cancers. While the overexpression of SWELL1 resulted in the opposite results in the cells (**Figure S3B-C**). Interestingly, we demonstrated that si-SWELL1 was able to abolish the ethanol-mediated increase of triglyceride and cholesterol in HepG2.2.15 cells (**Figure 3G-H**), suggesting that SWELL1 is responsible for the event that ethanol modulates abnormal lipid metabolism in the cells. However, we found that knocking down of SWELL1 by si-SWELL1 failed to reduce the lipid contents in ethanol-treated HBV-free HepG2 cells (**Figure S3D-E**), implying that the chronic ethanol consumption induce abnormal lipid metabolism through SWELL1 in a HBV-dependent manner. Meanwhile, the efficiency of HBx siRNA in HepG2.2.15 cells and the efficiency of SWELL1 siRNA (or SWELL1 overexpression) in three cell lines were confirmed by RT-qPCR (**Figure S3F-I**). Thus, we conclude that the ethanol enhances abnormal lipid metabolism through HBx/SWELL1 signaling.

### SWELL1 modulates arachidonic acid metabolism signaling *in vivo* and *in vitro*

**Figure 4:**
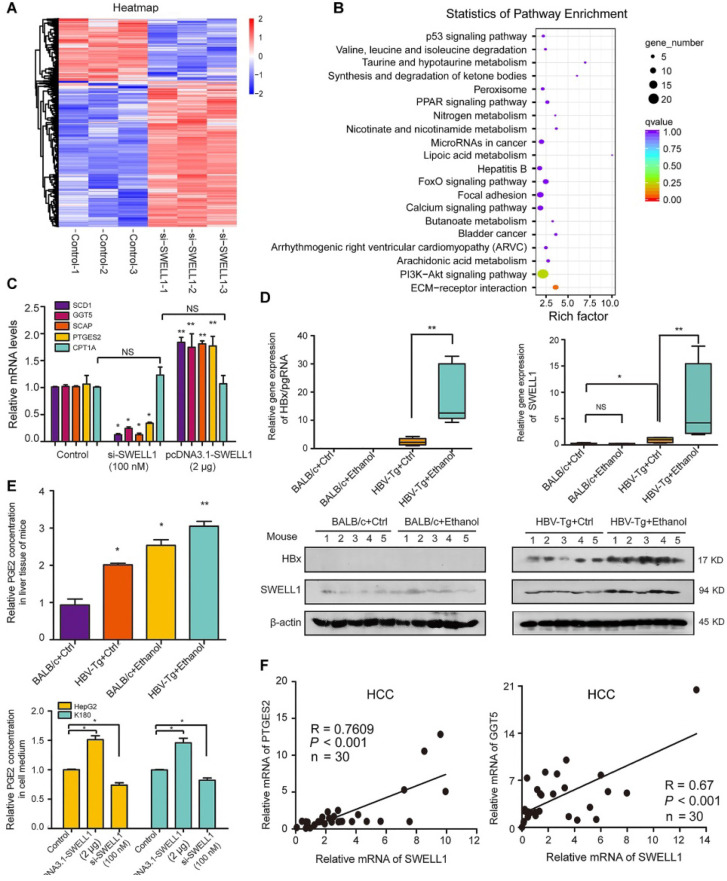
** SWELL1 modulates arachidonic acid metabolism signaling *in vivo* and *in vitro.*** (**A**) Heatmap displaying one-way hierarchical clustering and DEGs in control (n = 3) and si-SWELL1 (n = 3) in HepG2 cells across RNA-seq analysis. Up- and down-regulated genes were represented in red and blue colors, respectively. The color scale indicates at least 1.5 (log2) fold changes of the normalized hybridization signal intensities of genes. (**B**) Gene Set Enrichment Analysis revealing pathways and processes negatively enriched in si-SWELL1 compared with control in HepG2 cells. Q value is the FDR-adjusted p value. Q < 0.05 is considered statistically significant. (**C**) The mRNA levels of SCD1, GGT5, SCAP, PTGES2 and CPT1A were determined by RT-qPCR in HepG2 cells, respectively. (**D**) The mRNA levels and the protein levels of HBx and SWELL1 were assessed by RT-qPCR and Western blot analysis in the liver of each mice group, respectively. (**E**) Top panel: The relative levels of arachidonic acids metabolite PGE2 were detected by ELISA in the liver of each mice group. Bottom panel: The relative levels of PGE2 were detected by ELISA in cellular supernatant of HepG2 cells or K180 cells, respectively. (**F**) Correlation of mRNA levels between PTGES2 or GGT5 and SWELL1 was examined by RT-qPCR in 30 HCC clinical tissues. Each experiment was repeated at least three times. Error bars represent means ± SD (n = 3). *P < 0.05; **P < 0.01; NS, no significant; Student's *t*-test.

It has been reported that gamma-glutamyl transferase 5 (GGT5), a key metabolism component responsible for the catalysis of important anti-oxidant glutathione, can convert leukotriene C4 (LTC4, a glutathione S-conjugate) to LTD4 37. PTGES2 is involved in the synthesis of important arachidonic acid metabolite PGE2 13. To obtain unbiased insight into the putative SWELL1-dependent molecular pathways impacting lipid metabolism, we performed the analysis of RNA sequencing (RNA-seq) by using SWELL1 siRNA in HepG2 cells. The significant differences in RNA transcriptional profile were 932 differentially expressed genes (DEGs), in which 651 genes were up-regulated and 281 genes were down-regulated when SWELL1 was knocked down by SWELL1 siRNA in the cells (**Figure 4A and Figure S4A**). Interestingly, both Gene Ontology (GO) function enrichment analysis and Kyoto Encyclopedia of Genes and Genomes (KEGG) pathway analysis of the RNA-seq data demonstrated that SWELL1 might function through arachidonic acid pathway (**Figure 4B and Figure S4B**). Interestingly, GGT5 and PTGES2 were detectable in our list of DEGs.

Given that sterol regulatory element-binding transcription factor 1 (SREBF1)/SREBP cleavage-activating protein (SCAP) played crucial roles in modulation of abnormal lipid metabolism 38, we further analyzed fourteen lipid related genes mediated by si-SWELL1, including SREBP1-SCD1 axis, arachidonic acid metabolism related enzyme GGT5 and PTGES2 (**Figure S4C**). In addition, the interaction of SWELL1 with other molecules including lncRNA, miRNA and protein was demonstrated by using three databases (PPI, Starbase, LncRNADisease). The results were visualized on a network of gene sets connected by randomly distributed (**Figure S4D**). The overexpression of SWELL1 resulted in the up-regulation of SCD1, GGT5, SCAP and PTGES2 in HepG2 cells, whereas si-RNA led to the opposite data, and carnitine palmitoyltransferase 1A (CPT1A) related to the fatty acid oxidation was used as the negative control (**Figure 4C**). Next, we observed that the expression levels of GGT5, SCD1 and SCAP were significantly increased in PHH with *de novo* HBV infection (**Figure S4E**).

Next, the effect of chronic ethanol consumption on HBx-mediated activation of SWELL1 was verified in mice as above. In the liver of HBV-Tg mice fed with ethanol liquid diet, the mRNA and protein levels of HBx and SWELL1 were remarkably increased compared with the other three groups. However, we failed to observe the up-regulation of SWELL1 in normal mice fed ethanol liquid diets (**Figure 4D**). Accordingly 13, we showed that the levels of arachidonic acid metabolite PGE2 and/or LTD4 were both increased in the liver from HBV-Tg mice with ethanol (**Figure 4E,** top panel and**Figure S5A**). The overexpression of SWELL1 resulted in the increase of arachidonic acid metabolism in HepG2 cells or K180 cells by examining the amount of cellular PGE2 and LTD4 in cell medium, whereas si-RNA could lead to the opposite data (**Figure 4E,** bottom panel and**Figure S5B**), implying that SWELL1 is responsible for the modulation of arachidonic acid metabolism in the cells. As expected, we observed that the ethanol treatment enhanced the level of LTD4 in the supernatant of HepG2 and HepG2.2.15 cells, in which the levels of LTD4 were higher in HepG2.2.15 cells than those of LTD4 in HepG2 cells (**Figure S5C**). Consistently, the mRNA levels of GGT5 and PTGES2 were remarkably increased in ethanol-fed HBV-Tg mice, relative to other three groups (**Figure S5D-E**). Clinically, we validated that the expression levels of SWELL1 were significantly associated with those of PTGES2 (Pearson correlation: R = 0.7609) or GGT5 (R = 0.67) or SCD1 (R = 0.8103) in HCC tissues (**Figure 4F and Figure S5F**). Thus, we conclude that SWELL1 modulates arachidonic acid metabolism signaling *in vivo* and *in vitro.*

### Ethanol intake increases PBL Treg population and splenic Treg population in HBV-Tg mice

**Figure 5:**
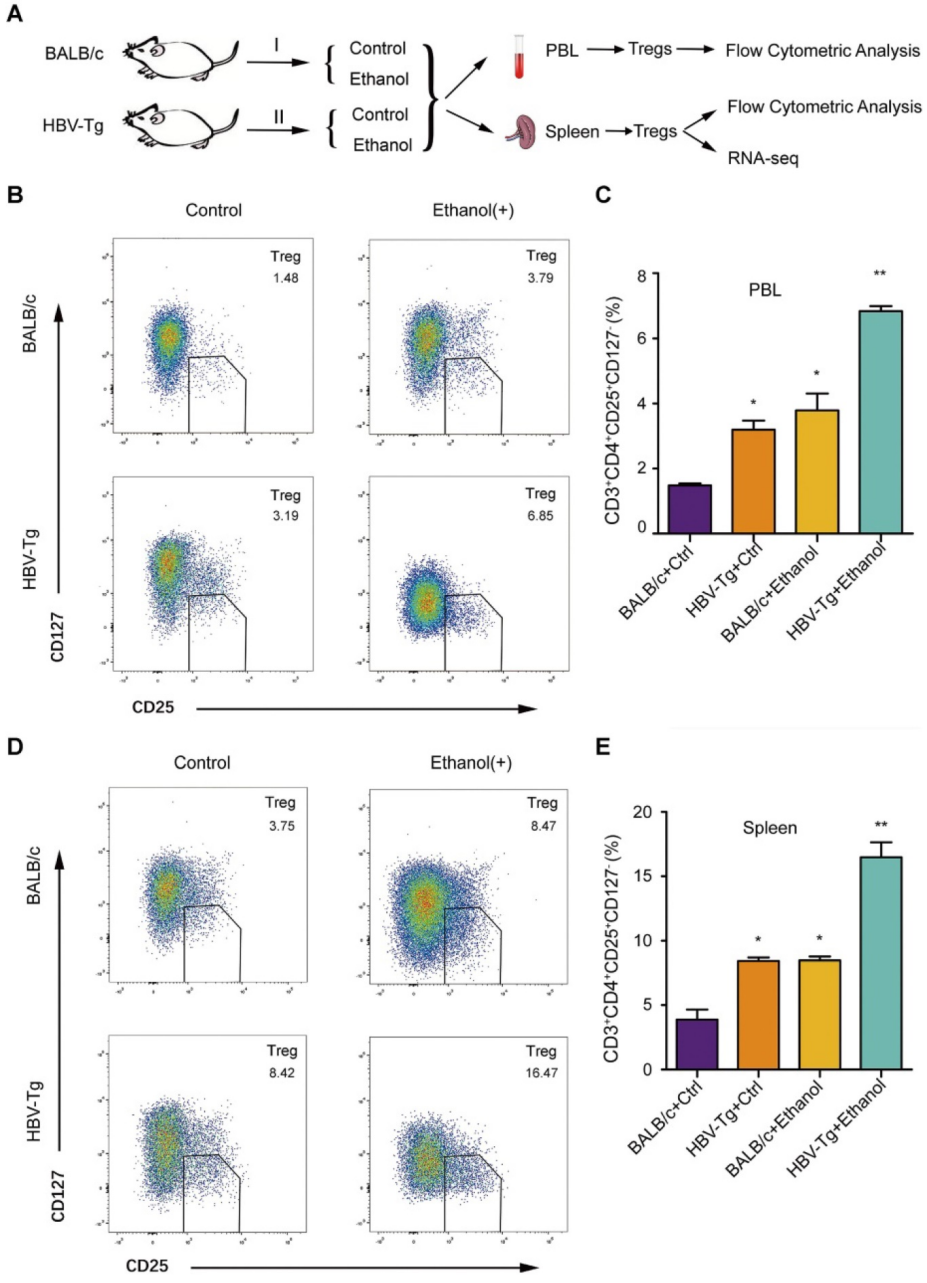
** Ethanol intake increases PBL Treg population and splenic Treg population in HBV-Tg mice.** (**A**) The strategy for studying the effect of chronic ethanol consumption on PBL and splenic Tregs in mice. (**B and D**) A composite flowcytometry panel showing gating strategy for the representative plot for CD3^+^CD4^+^CD25^+^CD127^-^ Tregs from PBL or spleen of each groups of mice. (**C and E**) The percentage of Tregs in PBL or spleen of each groups of mice. Error bars represent means ± SD (n = 3). Statistically significant differences are indicated: *P < 0.05, **P < 0.01; Student's *t*-test.

It has been reported that the ethanol-caused elevated-Tregs are of great significance 39. However, the effect of ethanol on Tregs is not well documented when the liver is infected by HBV. In this study, we examined the peripheral blood Treg in a model of HBV-Tg mice by flow cytometric analysis, including four groups: BALB/c mice fed with control normal liquid diet, BALB/c mice fed with ethanol liquid diet, HBV-Tg mice fed with control normal liquid diet and HBV-Tg mice fed with ethanol liquid diet (**Figure 5A**). Forkhead box P3 (FoxP3) is located in the intracellular region 40, and its detection requires intracellular staining, so that the cells were fixed and permeabilized using a specialized kit 41. It may bring a lot of inconvenience to the subsequent Treg sorting and preservation for RNA-seq. CD127 expression inversely correlates with FoxP3, then the Treg cells were defined as CD3^+^CD4^+^CD25^+^CD127^-^ cells, even though FoxP3 is an important marker of Treg cells 42, 43. Flow cytometric analysis indicated that the peripheral blood lymphocytes (PBL) isolated from ethanol-fed mice (or HBV-Tg mice) exhibited higher CD3^+^CD4^+^CD25^+^CD127^-^ Treg population, respectively, in comparison with normal mice (**Figure 5B**). The frequency of Tregs as a percentage of total peripheral blood lymphocytes in ethanol fed mice (3.79% ± 0.89) or HBV-Tg mice (3.19% ± 0.49) was significantly higher than that in BALB/c mice (1.48% ± 0.11). Moreover, ethanol intake significantly increased the magnitude of Tregs from 3.79% to 6.85% in HBV-Tg mice (**Figure 5C**). Meanwhile, the population of splenic Tregs was determined by flow cytometric analysis in the system. Interestingly, flow cytometric analysis indicated that the splenocytes isolated from ethanol-fed mice (or HBV-Tg mice) displayed a significantly higher CD3^+^CD4^+^CD25^+^CD127^-^ Treg population, compared with normal mice (**Figure 5D**). The gate for CD3^+^CD4^+^CD25^+^CD127^-^ Treg cells was set, the frequency of Tregs as a percentage of total spleen cells in the ethanol fed mice (8.47% ± 0.52) or HBV-Tg mice (8.42% ± 0.51) was significantly higher than that in control BALB/c mice (3.75% ± 1.22). Notably, ethanol intake significantly increased the magnitude of Tregs from 8.42% to 16.47% in HBV-Tg mice (**Figure 5E**). Thus, we conclude that the chronic ethanol intake promotes PBL Treg population and splenic Treg population in HBV-Tg mice.

### Profiling of Tregs induced by chronic ethanol consumption or HBV in mice

**Figure 6:**
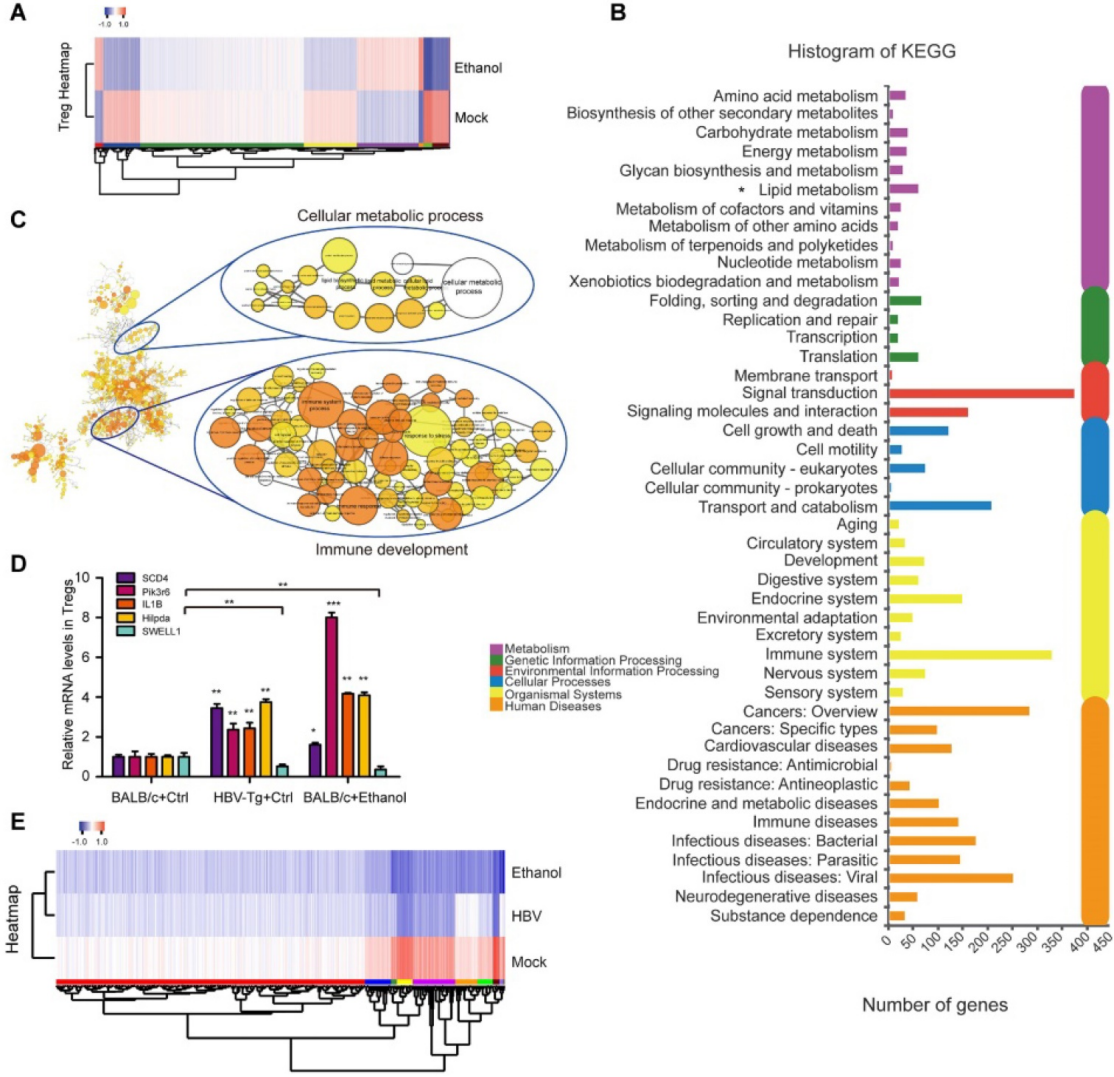
** Profiling of Tregs induced by chronic ethanol consumption or HBV in mice.** (**A**) Heatmap displaying hierarchical clustering and DEGs in splenic Tregs sorted from mock mice and ethanol-fed mice through RNA-seq analysis. Up- and down-regulated genes were represented in red and blue colors, respectively. The color scale indicates at least 2 (log2) fold changes of the normalized hybridization signal intensities of genes. (**B**) KEGG pathway analysis revealing pathways and processes enriched in DEGs of HBV-Tg group. (**C**) GO enrichment analysis of the ethanol-induced DEGs. The up-regulated genes were analyzed for GO term enrichment by gene-set enrichment analysis. The results were visualized on a network of gene sets (nodes) connected by their similarity (edges). The details were amplified in Figure S8. (**D**) The mRNA levels of SCD4, pik3r6, IL1B, Hilpda and SWELL1 were determined by RT-qPCR in Tregs sorted from mock mice, HBV-Tg mice and ethanol-fed mice, respectively. Error bars represent means ± SD (n = 3). *P < 0.05; **P < 0.01; ***P < 0.001; Student's *t*-test. (**E**) Heatmap displaying hierarchical clustering and DEGs of overlapping protein coed by SWELL1. Up- and down-regulated genes were represented in red and blue colors, respectively. The colour scale indicates at least 2 (log2) fold changes of the normalized hybridization signal intensities of genes.

The underlying mechanism for the effect of ethanol intake on Tregs is a mysterious issue. To identify the potential participators in the event, we delineated the profiling of splenic Treg heterogeneity by RNA-seq. Surprisingly, after Treg sorting we identified the transcriptomic atlas of 4907 genes (or 5558 genes) from ethanol fed mice group (or HBV-Tg mice group), relative to the BALB/c mice group fed with normal liquid diet as the mock group (**Figure 6A and Figure S6A**). Moreover, KEGG pathway analysis of the RNA-seq data demonstrated that the DEGs in Tregs of HBV-Tg mice may play a variety of roles involved in lipid metabolism (**Figure 6B**), consistent with the data in ethanol fed mice (**Figure S6B**). In the profiling of RNA-seq, the expression levels of the surface markers were not changed after ethanol consumption, including CD3, CD4, CD25 and CD127. We performed gene-set enrichment analysis of up-regulated genes in ethanol-fed group followed by network visualization (**Figure 6C**). Two major groups of clustered networks emerged. One was enriched for immune development-relevant GO terms such as immune system process and inflammatory response. The other was enriched for cellular metabolic process-relevant GO terms including lipid metabolism and several other metabolic processes. The details of **Figure 6C** were amplified in **Figure S8**.

In agreement with this, further analyses of membrane protein in Tregs showed that lipid metabolic proteins were up-regulated in the Tregs sorted from ethanol-fed mice, such as Hilpda (**Figure S6C-D**). Considering that membrane proteins play critical roles in facilitating the efficient generation of Tregs 44, 45, we were interested in the alteration of membrane proteins of Tregs in our system. Strikingly, we found that the expression levels of 715 membrane proteins were remarkably up-regulated, such as SCD4 and pik3r6, in Tregs induced by ethanol as shown in **Supplementary File 1**, implying that the up-regulated membrane proteins in Tregs may be involved in the event of immune response. As expected, we observed that the expression levels of SCD4, pik3r6, IL1B and Hilpda were significantly increased in the Tregs sorted from HBV-Tg mice and ethanol-fed mice, while the opposite trend was obtained in the levels of SWELL1 (**Figure 6D**). SWELL1 plays an important role in T cell development and adipocyte metabolic function 27. The interactions among the altered genes involving SWELL1 were analyzed as shown in **Figure 6E and Figure S7A**. And the expression levels of SWELL1 related DEGs in Tregs sorted from HBV-Tg mice and ethanol-fed mice were shown in **Supplementary File 2**. Thus, we conclude that the chronic ethanol consumption results in profiling alteration in Tregs, presenting the effect of chronic ethanol consumption on the immune molecular response of Tregs in mice.

## Discussion

A great portion of CHB patients are suffering from concomitant ALD, which is a public health problem worldwide. It has been reported that chronic ethanol consumption disrupts cholesterol homeostasis 10, and ethanol-caused elevated-Tregs are of great significance 39. However, the significance of ethanol in the moudulation of HBV-induced abnormal lipid and Tregs is elusive. In this study, we investigated the combination effect of chronic ethanol consumption with HBV on abnormal lipid metabolism and Tregs in a model of HBV-Tg mice.

Given that the chronic ethanol consumption frequently enhanced the progression of abnormal lipid metabolism 10, we concerned the infulence of chronic ethanol consumption in the event. As expected, we found that the treatment remarkably induce the abnormal lipid metabolism deposition in liver infected with HBV. Our data suggest that the ethanol cooperatively enriches the role of HBV in the modulation of abnormal lipid metabolism. It has been reported that SWELL1 plays a role in the regulation of lipid content in normal adipocyte cells 28. Thereby, we concerned in the role of SWELL1 in modulating abnormal lipid metabolism in liver cancer cells. Clinically, the analysis of TCGA data revealed that SWELL1 was an important factor of evaluating the risk of mortality and DRG of HCC patients, and high levels of SWELL1 were related to the poor survival for HCC patients. Furthermore, we validated the role of SWELL1 in the modulation of lipid metabolic process in hepatoma and esophageal carcinoma. To better understand the underlying mechanism by which SWELL1 regulated abnormal lipid metabolism, we demonstrated the profiling of SWELL1 in hepatoma cells using RNA-seq. Interestingly, RNA-seq data showed that SWELL1 might modulate arachidonic metabolism signaling, lipoic acid metabolism, nitrogen metabolism, p53 signaling pathway and PI3K-Akt signaling pathway, etc. A SWELL1 downstream lipid related pathway was linked to GGT5, which functioned in the transformation of LTC4 to LTD4 in arachidonic acid metabolism 37. SREBP-1 and SCD1 are the central players in lipid metabolism 38. Moreover, we found that another SWELL1 downstream gene was PTGES2. Considering that PGE2 promotes Treg activities in human lymphocytes which contributes to the tumor-induced immunosuppression 13-15, our finding suggests that SWELL1 modulates abnormal lipid metabolism through arachidonic acid metabolism signaling, which might activate Tregs.

Coincidentally, HBx may promote proliferation of liver cells by altering the expression of genes that participated in arachidonic acid metabolism 46. Our group has reported that the integration of HBx fragment can be detected in clinical hepatocellular carcinoma tissues 31. It has been reported that the ethanol enhances transcriptional activity of HBV promoters in an oxidative stress-independent manner, and CYP2E1-mediated oxidative stress potentiated the ethanol-induced transactivation of HBV 5 and the ethanol potentiates HBx in liver 47-49. In this study, we validated that ethanol intake increased HBx expression* in vivo* and *in vitro*, and knocking down of HBx by si-HBx could block the ethanol-induced lipid droplet accumulation in HepG2.2.15 cells. It suggests that the chronic ethanol consumption and HBV induce abnormal lipid metabolism through HBx. HBx can induce fatty acid and cholesterol synthesis *via* transcriptional activation of SREBP1 50. In this study, we found that HBV increased the levels of SWELL1 in PHH and further identified that HBx up-regulated SWELL1 through co-activating transcription factor Sp1 to promote lipid accumulation *in vivo* and *in vitro*. Our data showed that the silencing SWELL1 blocked the ethanol-induced increase of lipid contents in HepG2.2.15 cells, rather than in HBV-free HepG2 cells. It suggests that SWELL1 is responsible for the event that ethanol modulates aberrant lipid metabolism only in hepatoma cells with HBV. Therefore, the chronic ethanol consumption and HBV induce abnormal lipid metabolism through HBx/SWELL1/arachidonic acid signaling. Therapeutically, SWELL1 is potential target for abnormal lipid metabolism in liver.

The host nutritional status, including the uptake and generation of various metabolites, can modulate Treg abundance and function 51. Collectively, our data suggest that the chronic ethanol intake impacts peripheral Treg homeostasis. Interestingly, we observed that the peripheral blood and splenocytes isolated from ethanol-fed HBV-Tg mice exhibited a significantly higher CD3^+^CD4^+^CD25^+^CD127^-^ Treg population, relative to control group. It suggests that the chronic alcoholic consumption is able to elevate the HBV-induced Treg population *in vivo*. Since it is not clearly defined that the nature of circulating Tregs during chronic ethanol intake in HBV infection condition, we hypothesized that the enhancement of Treg population might represent a hallmark for inappropriate adaptive immune response during chronic liver damage caused by ethanol. Recent research has reported that the alcohol is able to increase the frequency of Tregs in patients and results in metabolic dysregulation 52, but the participators involved in this event need further investigation. Because the clinical experiment of chronic ethanol consumption on HBV patients is an ethical issue, to identify the potencial regulators and figure out how they are wired together, we systematically detected the synergistical effect of choronic ethanol consumption and HBV on lipid metabolic disorder and concequently Treg response in a model of HBV-Tg mice.

Up to now, the profiling of Tregs has not been reported yet. To comprehensively delineate the Treg heterogeneity under different group and the underlying mechanism for Treg progression, we analyzed the transcription status of Tregs sorted from each group on transcriptional profile by using RNA-seq analysis. Amazingly, we acquired the transcriptomic atlas of 4907 genes (or 5558 genes) by Treg sorting from spleen of ethanol-fed group (or HBV-Tg mice), relative to mock group, respectively. The roles of DEGs involve lipid metabolism, amino acid metabolism, cell growth and death, carbohydrate metabolism and signal transduction, etc. Basically, Treg is expressing different immune checkpoint molecules favoring immune suppressive function. Immune checkpoint molecules are coinhibitory receptors observed on the surface of multiple immune cells. After binding to specific ligands, these molecules can transmit inhibitory signals into cells to negatively modulate the immune response. Their classical physiological function is to inhibit immune cell-mediated inflammatory response to prevent an overactivated immune attack. Therefore, these molecules are considered actively contribute to the induction of tumor immune escape, and autoimmunity prevention. However, the modulatory effects of immune checkpoint molecules on Treg cells during metabolic disorder mediated by ethanol are still largely unknown 44. Based on that the membrane proteins played important roles in facilitating efficient generation of Tregs, we are interested in the membrane proteins of Tregs. Strikingly, we found that the expression levels of 715 membrane proteins, including CD74, CD36 and Alox15, were remarkably up-regulated in Tregs induced by chronic ethanol intake in mice. According to the transcriptional profile, we analyzed the effects of chronic ethanol consumption on the factors that participated immune response, lipid metabolism and other pathways. It has been reported that SCD4, pik3r6 and Hilpda are highly relevant in modulating lipid metabolic in the cells 53-56, but their roles in Tregs remains unclear. The decreased IL-1β-induced CCL20 secretion by schizophrenia iPSC-astrocytes cause potential attenuating effects on recruitment of Tregs. Paeoniflorin regulates the function of human peripheral blood mononuclear cells stimulated by rhIL-1β by up-regulating Treg expression 57. In this study, we found that the expression levels of SCD4, pik3r6; IL1B and Hilpda were significantly increased in the Tregs sorted from HBV-Tg mice and ethanol-fed mice. Thus, the finding those ethanol up-regulated membrane proteins in Tregs may provide new potential immune therapeutic targets. Based on the Treg transcriptional profile, we need to determine the effects of ethanol on Treg function with respect to the regulation of antigen specific immune responses in the future study.

Given that PD-L1 expression by tumor cells was strongly correlated with tumor immune escape 18, thus we were interested in the effect of ethanol intake on PD-L1 in liver. Interestingly, our data showed that the expression levels of PD-L1 from liver were significantly increased in ethanol-fed mice and HBV-Tg mice, but the most increased levels of PD-L1 were observed in the ethanol consumption with HBV mice compared with pair-fed mice (**Figure S7B-C**). Consistently, the treatment with ethanol was capable of elevating mRNA levels of PD-L1 in a dose-dependent manner in hepatoma cells, and the range was especially higher in HBV-expressing HepG2.2.15 cells (**Figure S7B**, left panel). Clinically, based on that the ethanol enhances the transcriptional activity of HBV promoters and induces HBV reactivation 5, 7, our data imply that the resistance of immune checkpoint inhibitors might be involved in the event due to the up-regulation of PD-L1 mediated by ethanol. Considering that the inhibitory signaling is transmitted by the formation of PD-L1 and its receptor PD-1, inducing T cell tolerance 16, the chronic ethanol intake may enhance the risk for immune deficiency in liver diseases caused by HBV infection clinically. High PD-L1 expression negatively impacted the prognosis of patients with HBV infection and PD1/PD-L1 immune checkpoint inhibitors showed promising results for several malignancies 58. Potentially, our finding suggests that the infection of HBV and chronic ethanol consumption might result in the resistance of immune checkpoint inhibitors because HBV infection and ethanol up-regulate PD-L1 in liver.

The key role of Tregs in metabolic disorders is highlighted recently, and the generation and immunosuppressive functions of Tregs are influenced by both systemic and cellular metabolism. Apart from lipid content, in this study we found that the chronic ethanol intake and HBV infection could elevate the Treg population and the expression of effector molecules PD-L1 in liver. The nutritional status as well as arachidonic acid metabolite, hence cueing impinge upon the proliferation of Tregs. Usually, Treg is activated by excessive lipid content which regulates the balance between protective immunity and host immune-mediated damage 12, 51. Strikingly, we found that ethanol and SWELL1 could elevate the levels of PGE2 or LTD4* in vivo* and *in vitro*. It strongly suggests that the ethanol or SWELL1-elevated PGE2 or LTD4 might enhance Treg population, which is consistent with that PGE2 is responsible for the activation of Tregs 13. Thus, we conclude that the chronic ethanol consumption enriches the HBV-induced abnormal lipid metabolism and activates Tregs in mice.

**Figure 7:**
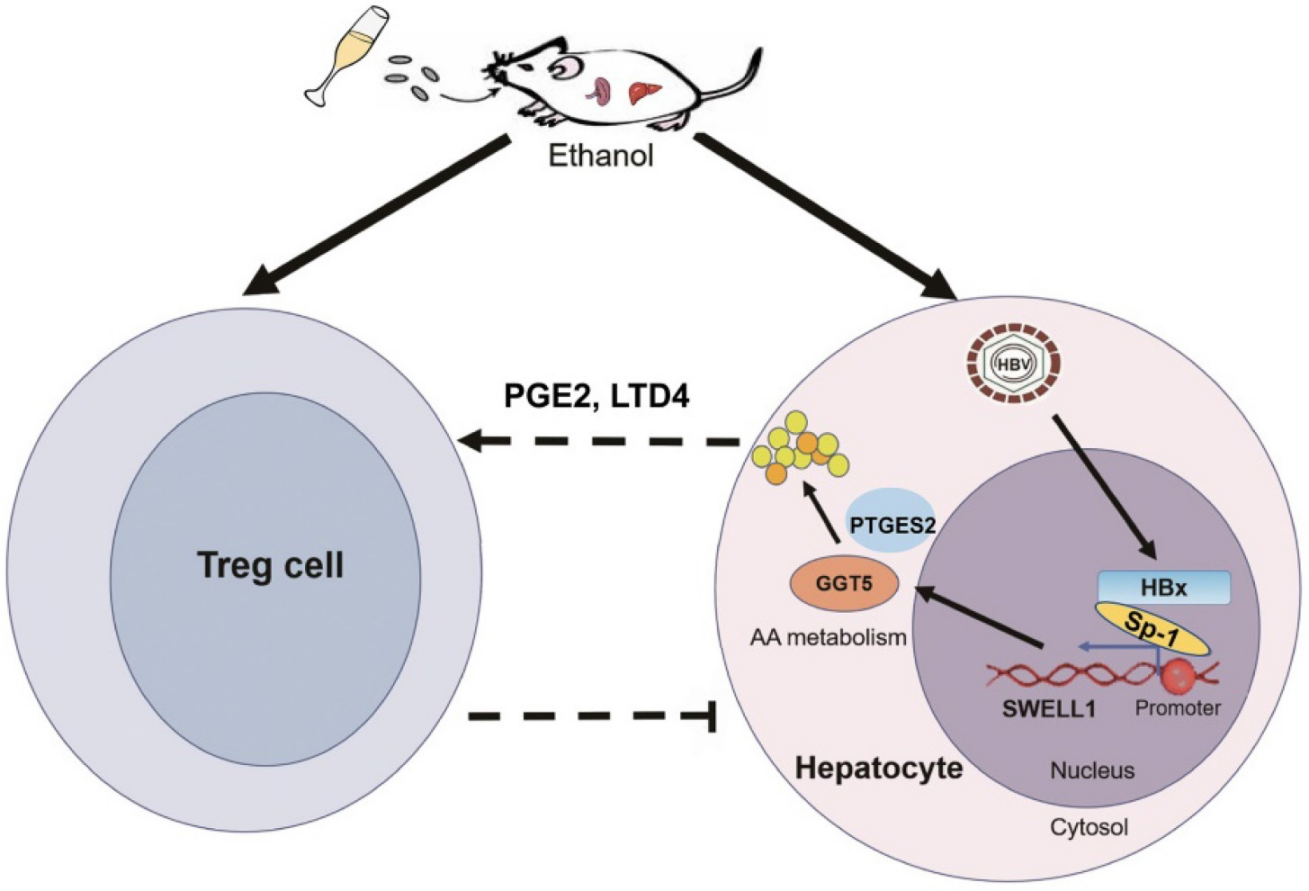
** A model of chronic ethanol consumption along with HBV in modulation of abnormal lipid metabolism and Treg in mice.** The chronic ethanol consumption and HBV cooperatively trigger abnormal lipid metabolism through HBx/Sp1/SWELL1/arachidonic acid metabolism signaling in liver. The ethanol or SWELL1-elevated PGE2 or LTD4 potentially enhances Treg population. The cooperative effects of chronic ethanol consumption and HBV on promoting abnormal lipid metabolism and activating Tregs is indicated in liver.

In summary, we employed a model of chronic ethanol consumption along with HBV expression in the modulation of abnormal lipid metabolism and Tregs in HBV-Tg mice (**Figure 7**). In this model, the chronic ethanol consumption and HBV cooperatively trigger the abnormal lipid metabolism through HBx/Sp1/SWELL1/arachidonic acid metabolism signaling in liver. Then, the ethanol or SWELL1-elevated PGE2 or LTD4 potentially enhances Treg population. Thus, the consequent induction of cooperative effects of chronic ethanol consumption and HBV on abnormal lipid metabolism and Tregs is demonstrated in liver.

## Materials and Methods

### RNA sequencing data

The RNA sequencing (RNA-seq) data reported in this paper have been deposited into sequence read archive (SRA) database under BioProject accession number PRJNA 560469 (mice Treg sequencing) and PRJNA 560496 (SWELL1 in liver cancer), respectively.

### Animals

HBV-Tg mice containing the intact HBV genome were obtained from VITALRIVER experiment animal company (Beijing, China). The generation, specific histological changes and Lieber-DeCarli liquid ethanol diet of HBV-Tg mice have been reported by our and other laboratory 20, 49, 59.

Mice were housed and treated according to the guidelines established by the National Institutes of Health Guide for the Care and Use of Laboratory Animals. The wild·type BALB/c mice were purchased from HFK bioscience company (Beijing, China) and housed in a temperature-, humidity-, and light-controlled room. Male mice were then randomly divided into four groups with an amount of 5 per group and were fed either a Lieber-DeCarli liquid ethanol diet or an isocaloric control diet, as described in previous studies 49, 59. Nontransgenic mice were fed an ethanol (group 1) or isocaloric diet (group 2). Likewise, transgenic mice were fed either an ethanol (group 3) or isocaloric diet (group 4). The wild type BALB/c mice and HBV-Tg mice were fed a nutritionally adequate DeCarli-Lieber liquid diet containing 5% ethanol for 4 weeks starting at the age of 6 weeks. We then switched them to the same diet containing 7% ethanol for additional 4 weeks. Weight-matched littermates were pairfed on the same liquid diet, except that ethanol was replaced by carbohydrate. Then, animals were sacrificed and liver, spleen and blood was collected. All animal procedures were performed according to the criteria outlined in the Guide for the Care and Use of Laboratory Animals (National Institutes of Health, Bethesda, MD, USA) and with approval of the Animal Care and Use Committee of Nankai University.

### Patient samples

Thirty HCC tissues utilized in this study were obtained from Tianjin Medical University Cancer Institute and Hospital (Tianjin, China) after surgical resection. Written consents approving the use of their tissues for research purposes after the operation were obtained from each patient. All study procedures were in compliance with the regulations of the institute of research ethics committee at Nankai University (Tianjin, China). The medical records of the patients were listed in **Table S1**.

### Cell lines and cell culture

The human hepatoma cell lines HepG2 and HepG2.2.15 were maintained in Dulbecco's modified Eagle's medium (Gibco, Grand Island, NY, USA) supplemented with 10% fetal bovine serum (FBS). The human esophageal cell line K180 was maintained in RPMI-1640 medium (Gibco, Grand Island, NY, USA) supplemented with 10% FBS. PHHs were purchased from Shanghai RILD Inc. (Shanghai, China). All the cell lines were treated with 100 U/mL penicillin, and 100 mg/mL streptomycin in 5% CO_2_ at 37 °C. The cells were cultured in a 6-well, 24-well or 96-well plate for 36 h or 48 h and then were transfected with plasmids or siRNAs. In ethanol-treated experiments, the cells were treated with 75 or 150 mM ethanol (Sigma-Aldrich, St.Louis, MO, USA) for 24 h in specific experiments, respectivily.

### RNA extraction and quantitative real-time PCR (RT-qPCR)

Total RNA was extracted from cells (or liver tissues from mice and patient tissues) using Trizol reagent (Invitrogen, Carlsbad, CA, USA) according to the manufacturer's protocol. First-strand cDNA was synthesized as reported previously. Reverse transcription was performed using ImPro-II Reverse Transcriptase (Promega, Madison, WI, USA), according to the manufacturer's instructions. RT-qPCR was performed by a Bio-Rad sequence detection system according to the manufacturer's instructions using double-stranded DNA-specific SYBR Green Premix Ex Taq™ II Kit (TaKaRa, Ohtsu, Japan). Experiments were conducted in duplicate in three independent assays. Relative transcriptional folds were calculated as 2^-∆∆Ct^. GAPDH was used as an internal control for normalization. All the primers used were listed in **Table S2**.

### RNA-seq

Total RNA was extracted using the trizol reagent, according to the manufacturer's instructions. RNA integrity and concentration were checked using NanoDrop ND-1000 spectrophotometer (Thermo Scientific, Wilmington, DE, USA) and an Agilent 2100 Bioanalyzer (Agilent Technologies, Santa Clara, CA, USA). mRNA was isolated by NEBNext Poly (A) mRNA Magnetic Isolation Module (NEB, E7490). The cDNA library was constructed following the manufacturer's instructions of NEBNext Ultra RNA Library Prep Kit for Illumina (NEB, E7530) and NEBNext Multiplex Oligos for Illumina (NEB, E7500). Briefly, the enriched mRNA was fragmented into approximately 200 nt RNA inserts, which were used to synthesize the first-strand cDNA and the second cDNA. The double-stranded cDNA were performed end-repair/dA-tail and adaptor ligation. The suitable fragments were isolated by Agencourt AMPure XP beads (Beckman Coulter, Inc.), and enriched by PCR amplification. Finally, the cDNA libraries of the cells were sequenced using an Illumina HiSeq™ 2500 sequencing platform. Then the prepared libraries were sequenced using an Illumina HiSeq 4000 sequencer (Biomarker Technologies, China). The RNA sequencing data (BioProject accession number PRJNA 560469 and PRJNA 560496) reported in this paper have been deposited into Sequence Read Archive (SRA) database. Hierarchical cluster, scatter plot analyses of gene expression levels and GSEA analysis were analyzed on the free online platform of Majorbio Cloud Platform (PRJNA 560469) and BMK Cloud Platform (PRJNA 560496), respectively. Pheatmap software was used in drawing the heatmap to determine the expression patterns of genes under different experimental conditions. Differential expression analysis of two groups was performed using the DESeq2. DESeq2 provide statistical routines for determining differential expression in digital gene expression data using a model based on the negative binomial distribution. The FDR < 0.05 and Fold Change ≥ 1.5 was set as the threshold for significantly differential expression in PRJNA 560496. And the FDR < 0.05 and Fold Change ≥ 2 was set as the threshold for significantly differential expression in PRJNA 560469. The clustering method adopted is h-cluster (complete algorithm). GO term enrichment was carried out as described, which was followed by network visualization in Cytoscape using the EnrichmentMap plugin.

### Cell transfection

The cells were cultured in a 6-well or 24-well plate for 24 h and then were transfected with plasmids or siRNAs. The transfections were performed using Lipofectamine 2000 reagent (Invitrogen, Carlsbad, CA, USA) or lipofectamine Messenger MaxTM Reagent for PHH (Invitrogen, Carlsbad, CA, USA) according to the manufacturer's protocol. siRNA oligonucleotides and a non-specific scrambled control, all siRNAs were synthesized by RiboBio (Guangzhou, China). The siRNA duplexes sequences used were as previous study 28, 35. All the siRNAs used were listed in **Table S3**.

### Construction and production of plasmids

The fragment containing the coding sequence (CDS) of SWELL1 and HBx was cloned into pcDNA3.1 vector. The primers used in this study for construction were listed in **Table S2**: various lengths of the SWELL1 5′ flanking region, including -1381/+215 (pGL3-SWELL1-promoter), -781/+215 (pGL3-F1), -600/+215 (pGL3-F2), -200/+215 (pGL3-F3), -1381/-781 (pGL3-SWELL1-promoter-CR-wt) and -1381/-781 (pGL3-SWELL1-promoter-CR-mut) were cloned, respectively. The recombinant vector pcDNA3.1 was used to overexpress SWELL1 in several cancer cell lines which were synthesized by RiboBio (Guangzhou, China).

### HBV preparation and infection

HBV used in this study was mainly derived from HepAD38 cells, which is classified as genotype D. HepAD38 cells were cultured in DMEM/F12 medium (Life Technologies, Carlsbad, CA, USA) supplemented with 10% heat-inactivated FBS, 100 U/mL penicillin, 100 μg/mL streptomycin, 100 μg/mL kanamycin, 400 μg/mL G418, and without any tetracycline (for induction of HBV replication, termed as HepAD38*). Media were recovered every 3 days from HepAD38* cells at day's 7-15 post-induction of HBV by depletion of tetracycline. Media were cleared through a 0.45 μm filter and precipitated with 10% PEG8000 and 2.3% NaCl. The precipitates were washed and resuspended with medium at 200-fold concentration 60, 61. The HBV DNA was quantified by RT-qPCR. PHH cells were infected with HBV at 500-1000 genome equivalents (GEq)/cell in the presence of 4% PEG8000 at 37 °C for 16 h as previously described 62.

### Western blot analysis

Western blot analysis protocol was described previously 63. Briefly, cellular proteins were extracted in RIPA buffer (Biomed, China) after transfection 48 h. Proteins were separated by gel electrophoresis and transformed to membranes, then membranes were blocked in 5% nonfat dry milk in 0.1% PBST for 2 h. Subsequently, primary antibody was diluted and incubated on the membranes at room temperature for 3 h. Next, membranes were treated with secondary antibody diluted in PBST at room temperature for 1 h. Then, they were washed in PBST and detected by enhanced chemiluminescence system plus western blotting detection reagents (Amersham Biosciences, Buckinghamshire, UK). β-actin was used as a loading control for Western blot analysis. All antibodies were listed in **Table S4**.

### Treg sorting

The splenocytes or PBL from mice were labelled with PerCP/Cy5.5‐conjugated CD3, PE-conjugated CD4, FITC-conjugated CD25 and APC-conjugated CD127 antibodies. The CD3^+^CD4^+^CD25^+^CD127^-^ cells were sorted with stringent gating conditions (BD FACSAria™ cell sorter); the sorted cells used for the experiments were 97-98% in purity, which was checked by using flow cytometry. Then the sorted Tregs were analyzed for phenotype and gene expression profiles. The antibodies used were listed in **Table S4**.

### Oil Red O staining and HE staining

Cells were seeded in 24-well plates and incubated overnight. After cells were treated with ethanol for 24 h or transfected with siRNA (plasmids) for 48 h, cells were washed twice with phosphate saline and fixed with 10% formalin. The Oil Red O staining was performed according to the manufacturer's instructions. For the HE staining, formalin fixed and paraffin embedded liver tissues were cut into 5 μm sections. Tissue slices were counterstained with hematoxylin-eosin (Biocare medical, China) according to the manufacturer's instructions, and dehydrated in ethanol, and then cleared in xylene (Fisher chemical, USA). Cover slips were added using histological mounting medium (Fisher, toluene solution, USA).

### HBsAg and HBeAg quantification

Secretion of HBsAg into the supernatants of cultured cells was measured by diagnostic kit for hepatitis B virus surface antigen according to the manufacturer's instructions (Kehua Bio-engineering, Shanghai, China). The cut-off value (COV) for HBsAg analysis was indicated as: COV = OD (negative control)/0.100. HBeAg in the supernatants of cultured cells was measured by diagnostic kit for Hepatitis B e antigen according to the manufacturer's instructions (Kehua Bio-engineering, Shanghai, China). The cut-off value for HBeAg analysis was indicated as: COV = OD (negative control)*2.1 (0 < ODNC ≤ 0.050, COV = 0.050*2.1 = 0.105; 0.050 < ODNC ≤ 0.100; ODNC > 0.100, invalidation).

### ELISA assays

For ELISA, the liver tissue of mice and supernatants from cell cultures (1 × 10^6^ cells/mL) were collected, and then concentrations of PGE2 or LTD4 were assessed using ELISA assay kit (Sinobestbio Technologies Inc., Shanghai, China). The levels of triglyceride or cholesterol in HepG2, HepG2.2.15 and K180 cells were assayed using cellular total triglyceride assay kit (Applygen Technologies Inc., Beijing, China) or cellular total cholesterol assay kit (Applygen Technologies Inc., Beijing, China). All the experiments were performed according to the manufacturer's recommended protocol.

### Luciferase reporter gene assays

Luciferase reporter gene assay was performed using the Dual-Luciferase Reporter Assay System (Promega, Madison, WI, USA) according to the manufacturer's instructions. Cells were transferred into 24-well plates at 3 × 10^4^ cells per well. After 24 h, the cells were transiently co-transfected with 0.2 μg/well of pRL-TK plasmid (Promega, Madison, WI, USA) containing the Renilla luciferase gene used for internal normalization, and various constructs containing different lengths of the SWELL1 5′-flanking promoter region and pGL3-basic as negative control. The luciferase activities were measured as previously described 20. All experiments were performed at least three times.

### Statistical analysis

Values are mean ± SEM excluding the data from RNA sequencing. Survival data were estimated using the Kaplan-Meier method, and differences in survival rates were compared using the log-rank test. Statistical analyses were performed using R software or Prism GraphPad Software version 4.0. One-way analysis of variance was performed to compare SWELL1 expression in all individual HBV-negative hepatoma cell lines with HBV-related hepatoma cell lines. Pearson's correlation coefficient was used to determine the correlation between SWELL1 and HBx/pgRNA levels in HCC tissues. All experiments were independently repeated at least three times. Significance values were set at *P < 0.05, **P < 0.01 and ***P < 0.001 (NS denotes nonsignificance, *P* > 0.05).

## Supplementary Material

Supplementary figures and tables.

Supplementary files.

## Abbreviations

HBV: hepatitis B virus
ALD: alcoholic liver disease
HCC: hepatocellular carcinoma
CLD: chronic liver disease
Tregs: regulatory T cells
PGE2: prostaglandin E2
PD-1: programmed death 1
PD-L1: programmed death ligand 1
CHB: chronic hepatitis B
HBx: HBV X protein
LRRC8A: leucine-rich repeat-containing protein 8A
PHH: primary human hepatocytes
siRNA: small interfering RNA
GGT5: gamma-glutamyl transferase 5
LTD4: leukotriene D4
RNA-seq: RNA sequencing
DEGs: differentially expressed genes
GO: gene ontology
KEGG: kyoto encyclopedia of genes and genomes
PBL: peripheral blood lymphocytes
mRNA: messenger RNA
RT-qPCR: quantitative real-time polymerase chain reaction
SD: standard deviation
