# Supplementary material for: Chronic ethanol consumption and HBV induce abnormal lipid metabolism through HBx/SWELL1/arachidonic acid signaling and activate Tregs in HBV-Tg mice
Source: Theranostics. 2020 Jul 23;10(20):9249–67. doi: 10.7150/thno.46005 (PMC7415795; doi:10.7150/thno.46005)
Supplement: Supplementary file 1 — Supplementary figures and tables. [file thnov10p9249s1.pdf]

Supplementary Figures

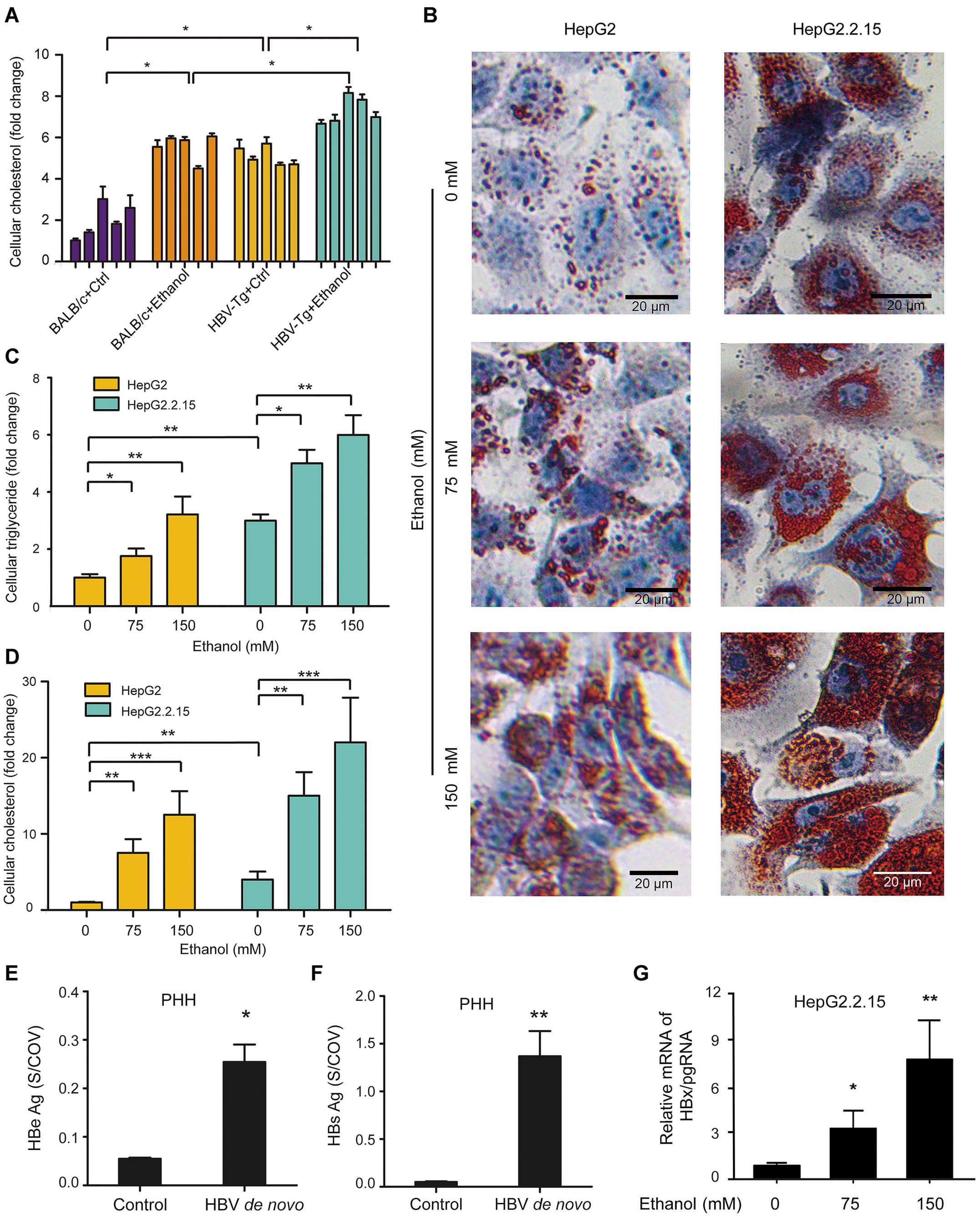

Figure S1 related to Figure 1. Ethanol enriches the HBV-enhanced abnormal lipid metabolism involving HBx and SWELL1. (A) The levels of cholesterol were individually measured in the liver tissues from four groups of mice (n = 5), including control BALB/c mice, BALB/c mice fed with ethanol, HBV transgenic mice and HBV transgenic mice fed with ethanol. (B) Oil Red O staining was used to examine the liquid droplets in HepG2 cells and HepG2.2.15 cells dose-dependently treated with ethanol. (C) The cellular triglyceride was measured by cellular

total triglyceride assay kit in HepG2 cells and HepG2.2.15 cells dose-dependently treated with ethanol. (D) The cellular cholesterol was detected by cellular total cholesterol assay kit in HepG2 cells and HepG2.2.15 cells dose-dependently treated with ethanol. (E) HBV indicated markers HBeAg were tested by ELISA assays in HBV-infected PHH cells. (F) HBV indicated markers HBsAg were tested by ELISA assays in HBV-infected PHH cells. (G) The effect of ethanol on mRNA levels of HBx/pgRNA was detected by RT-qPCR in HepG2.2.15 cells in a dose-dependent manner. All experiments were repeated at least three times. Error bars represent means ± SD (n = 3). Statistically significant differences are indicated: *$P < 0.05$, **$P < 0.01$, ***$P < 0.001$; Student's *t*-test.

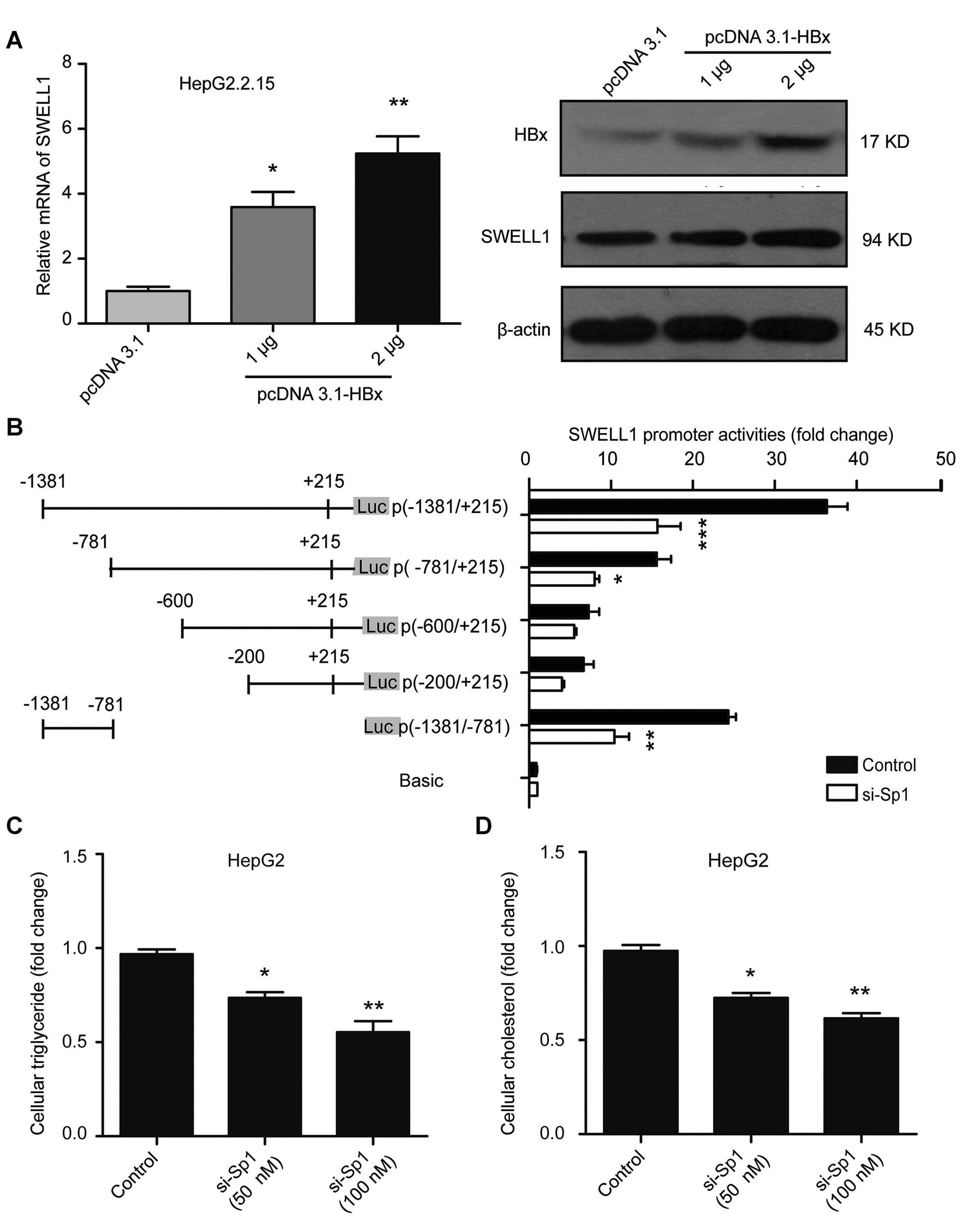

Figure S2 related to Figure 2. HBx up-regulates SWELL1 through co-activating transcription factor Sp1. (A) The mRNA and protein levels of SWELL1 were assessed by RT-qPCR and Western blot analysis in HepG2.2.15 cells transfected with pcDNA3.1-HBx (0, 1 μg, 2 μg), respectively. (B) HepG2 cells were transiently transfected with pGL3-Basic (0.2 μg/well) or reporter constructs containing various lengths of the 5′-flanking regions of the SWELL1 gene, as indicated (pGL3-SWELL1-promoter, pGL3-F1, pGL3-F2, pGL3-F3 and pGL3-SWELL1-CR-promoter respectively). Then, HepG2 cells were treated with 50 μM

si-Sp1 or 50 μM si-control, followed by luciferase reporter gene assays to examine the SWELL1 promoter activities. (C) The effect of Sp1 siRNA on cellular triglyceride was measured by cellular total triglyceride assay kit in HepG2 cells or the co-treatment of si-control, respectively. (D) The effect of Sp1 siRNA on cellular cholesterol was detected by cellular total cholesterol assay kit in HepG2 cells or the co-treatment of si-control, respectively. All experiments were repeated at least three times. Error bars represent means ± SD (n = 3). *$P < 0.05$, **$P < 0.01$, ***$P < 0.001$; Student's *t*-test.

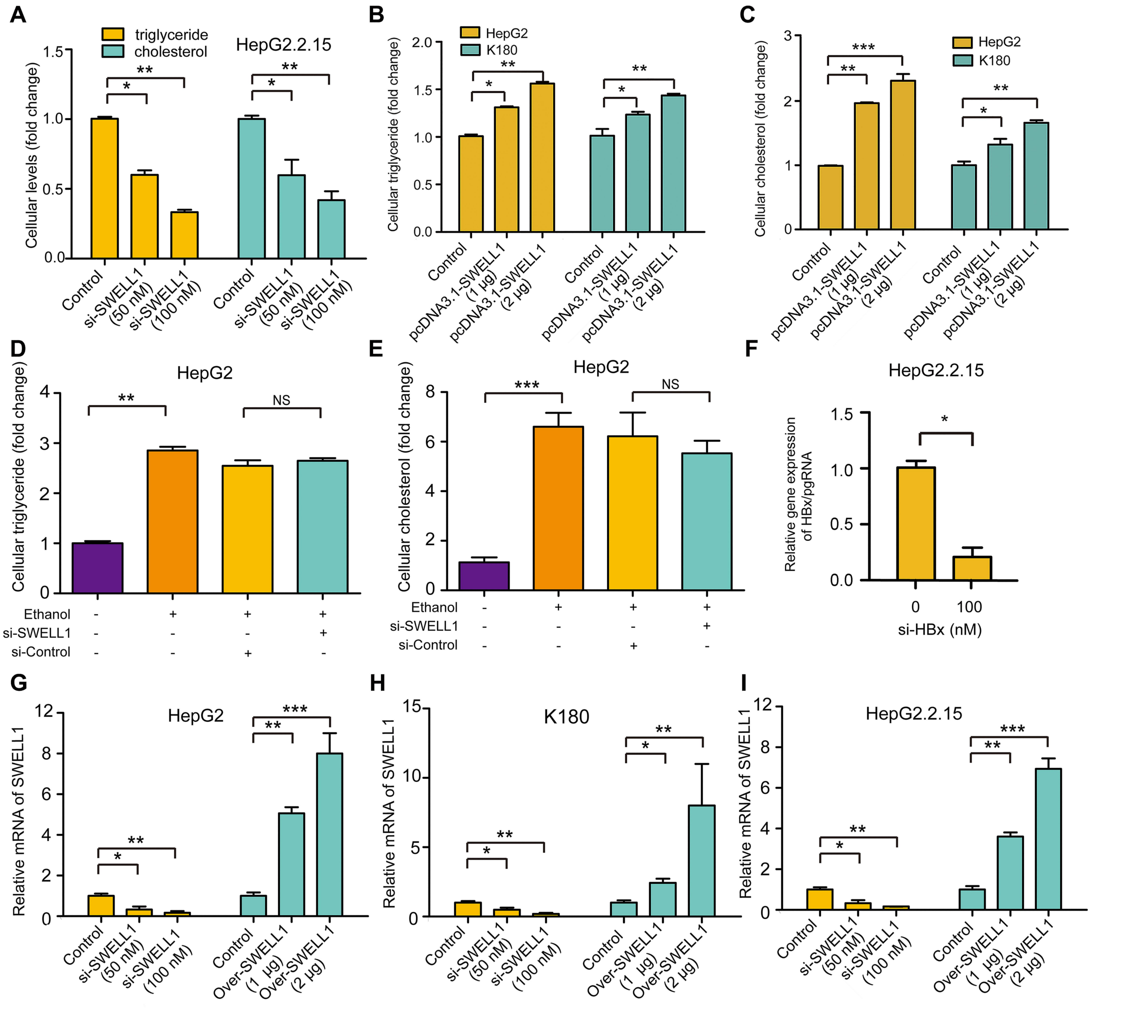

Figure S3 related to Figure 3. Ethanol enhances abnormal lipid metabolism through HBx/SWELL1 signaling. (A) The effects of SWELL1 siRNA on cellular triglyceride and cellular cholesterol were examined in HepG2.2.15 cells, respectively. (B) The effect of pcDNA3.1-SWELL1 on cellular triglyceride was detected by ELISA in HepG2 cells and K180 cells, respectively. (C) The effect of pcDNA3.1-SWELL1 on cellular cholesterol was detected by ELISA in HepG2 cells and K180 cells, respectively. (D) The effect of ethanol, SWELL1 siRNA or both on cellular triglyceride was measured by ELISA in HepG2 cells, respectively. (E) The effect of ethanol, SWELL1 siRNA or both on cellular cholesterol was detected by ELISA in HepG2 cells, respectively. (F) HBx siRNA (0, 100 nM) was transfected into HepG2.2.15 cells, in which the interference efficiency of HBx was detected by RT-qPCR in the cells. (G), (H) and (I) SWELL1 siRNA (0, 50 nM, 100 nM) was transfected into HepG2, K180 and HepG2.2.15 cells, in which the interference efficiency of SWELL was detected by

RT-qPCR in the cells, respectively. Meanwhile, pcDNA3.1-SWELL1 overexpression plasmid (0, 1 μg, 2 μg) were transfected into HepG2, K180 and HepG2.2.15 cells, in which the overexpression efficiency of SWELL was detected by RT-qPCR in the cells. All experiments were repeated at least three times. Error bars represent means ± SD (n = 3). $^{*}P < 0.05$, $^{**}P < 0.01$, $^{***}P < 0.001$; NS, no significant; Student's *t*-test.

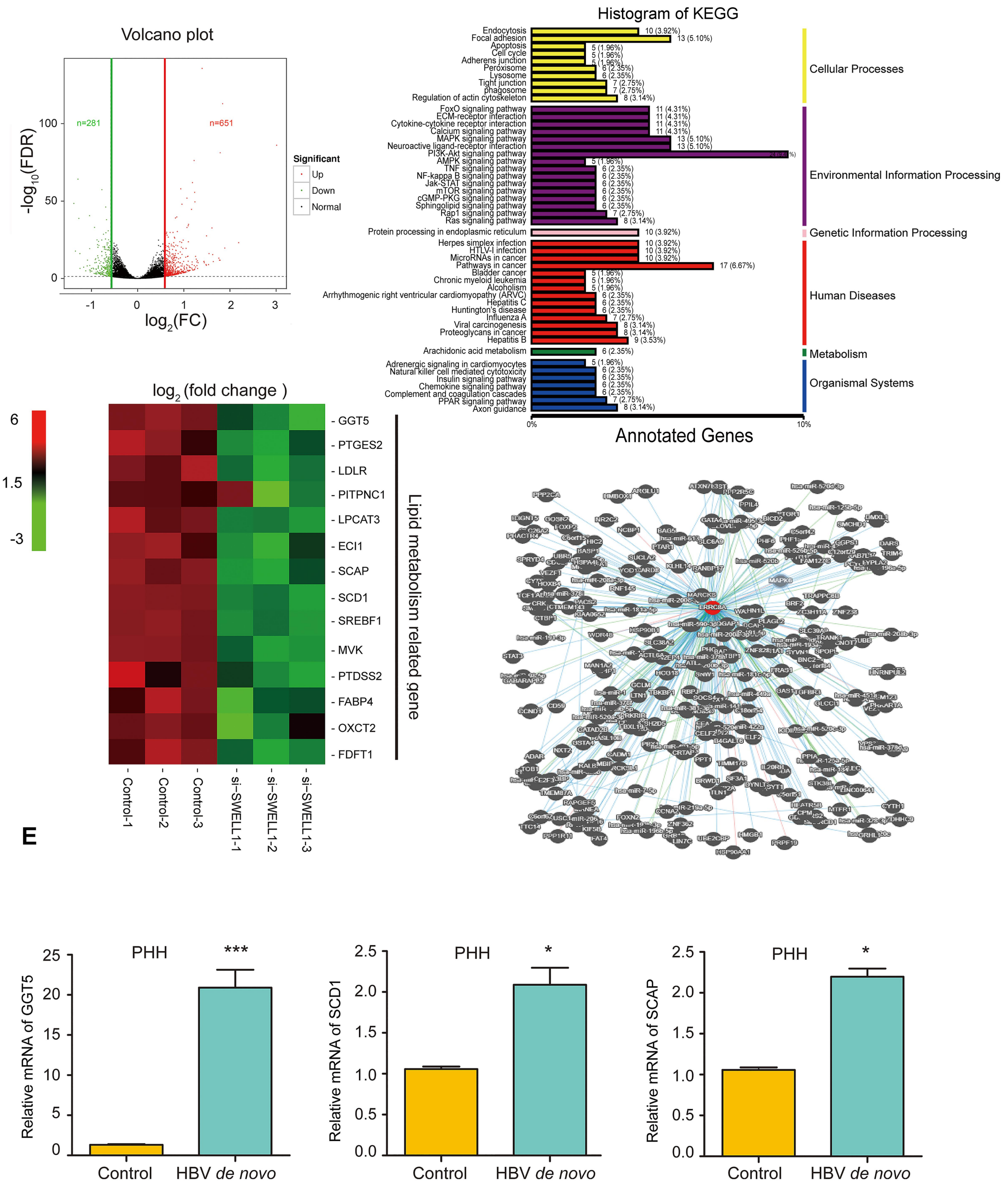

Figure S4 related to Figure 4. SWELL1 modulates arachidonic acid metabolism signaling *in vivo* and *in vitro*. (A) The DEGs was shown in volcano plot. Datasets from at least 3 independent biological replicates were shown. (B) Distribution of DEGs in representative pathways as compared between si-control or si-SWELL1 transfected HepG2. KEGG enrichment analysis was performed to identify pathways mediated by si-SWELL1. (C) Heatmaps depicted the relative fold change of gene expression based on the lipid pathway related genes. Heatmap shows the top fourteen genes that were down-regulated in HepG2

cells mediated by si-SWELL1. The web-based gene set analysis toolkit Web Gestalt was used to analyze a set of lipid metabolism related responding genes. Red and green indicate increased and decreased expression, respectively. (D) The interaction of SWELL1 with other molecules including lncRNA, miRNA and protein was demonstrated by using three databases (PPI, Starbase, LncRNA Disease). The analysis was based on the DEG list. (E) The mRNA levels of GGT5, SCD1, and SCAP were examined by RT-qPCR in *de novo* HBV-infected PHH cells, respectively. All experiments were repeated at least three times. Error bars represent means ± SD (n = 3). **P* < 0.05, ***P* < 0.01, ****P* < 0.001; Student's *t*-test.

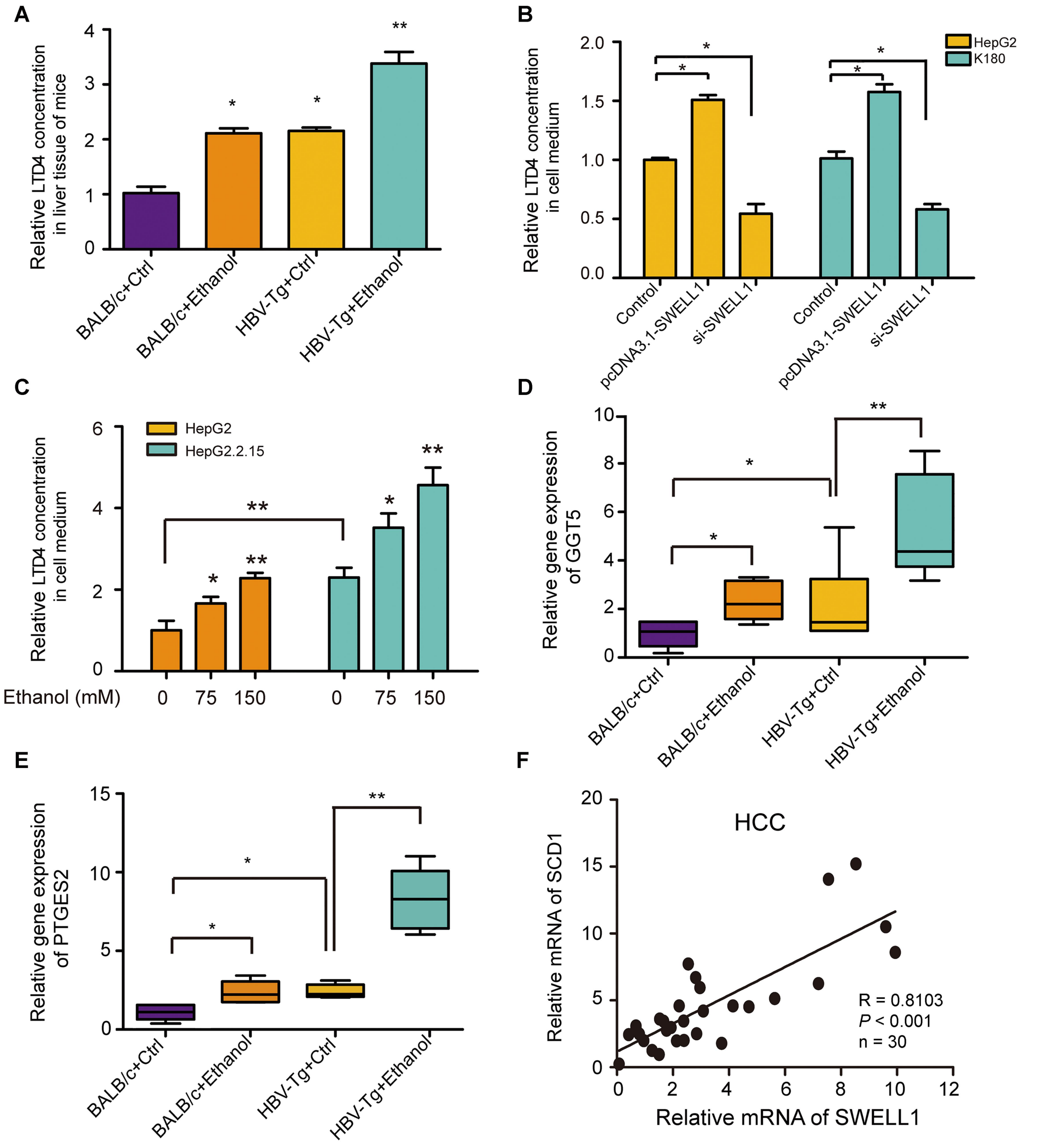

Figure S5 related to Figure 4. SWELL1 modulates arachidonic acid metabolism signaling *in vivo* and *in vitro*. (A) The relative levels of arachidonic acids metabolite LTD4 were detected in the liver of each mice group. (B) The relative levels of LTD4 were detected in cellular supernatant of HepG2 or K180 transfected with SWELL1 siRNA (100 nM) or pcDNA3.1-SWELL1 (2 μg), respectively. (C) The effect of ethanol on relative levels of LTD4 were detected in cellular supernatant of HepG2 or HepG2.2.15 in a dose-dependent manner. (D) and (E) The mRNA levels of GGT5 and PTGES2 were determined by RT-qPCR in each group of mice, respectively. (F) Correlation of mRNA levels between SCD1 and SWELL1 was examined by RT-qPCR in 30 clinical HCC tissues (R = 0.8103, P < 0.001,

Pearson's correlation coefficient). All experiments were repeated at least three times. Error bars represent means ± SD (n = 3). $^*P < 0.05$, $^{**}P < 0.01$; Student’s *t*-test.

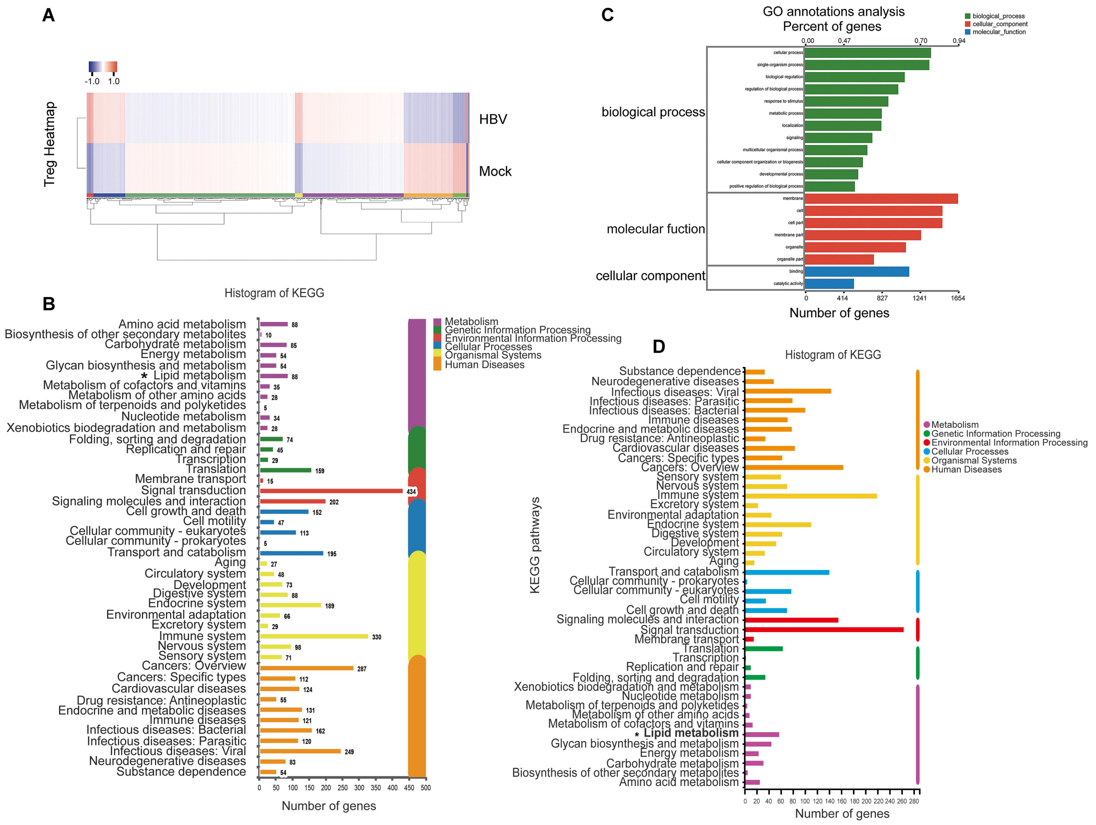

Figure S6 related to Figure 6. Profiling of Tregs induced by chronic ethanol consumption or HBV in mice. (A) Heatmap displaying hierarchical clustering and DEGs in splenic Tregs sorted from mock mice and HBV-Tg mice through RNA-seq analysis. Up- and down-regulated genes were represented in red and blue colors, respectively. The color scale indicates at least 2 (log2) fold changes of the normalized hybridization signal intensities of genes. Color key from blue to red indicates relative expression levels from low to high. (B) KEGG pathway analysis revealing pathways and processes enriched in DEGs of ethanol-fed mice group. (C) GO biological process analysis of the significant changes in splenic Tregs sorted from mock mice and ethanol-fed mice through RNA-seq analysis. Each annotated sequence was assigned at least one GO term in three main categories (biological process, cellular component and molecular function). The x-axis denotes the number of unigenes; the y-axis represents the GO term. (D) Distribution of DEGs of membrane protein in representative pathways as compared between splenic Tregs sorted from mock mice and Tregs sorted from ethanol-fed mice. KEGG enrichment analysis was performed to identify pathways mediated by ethanol.

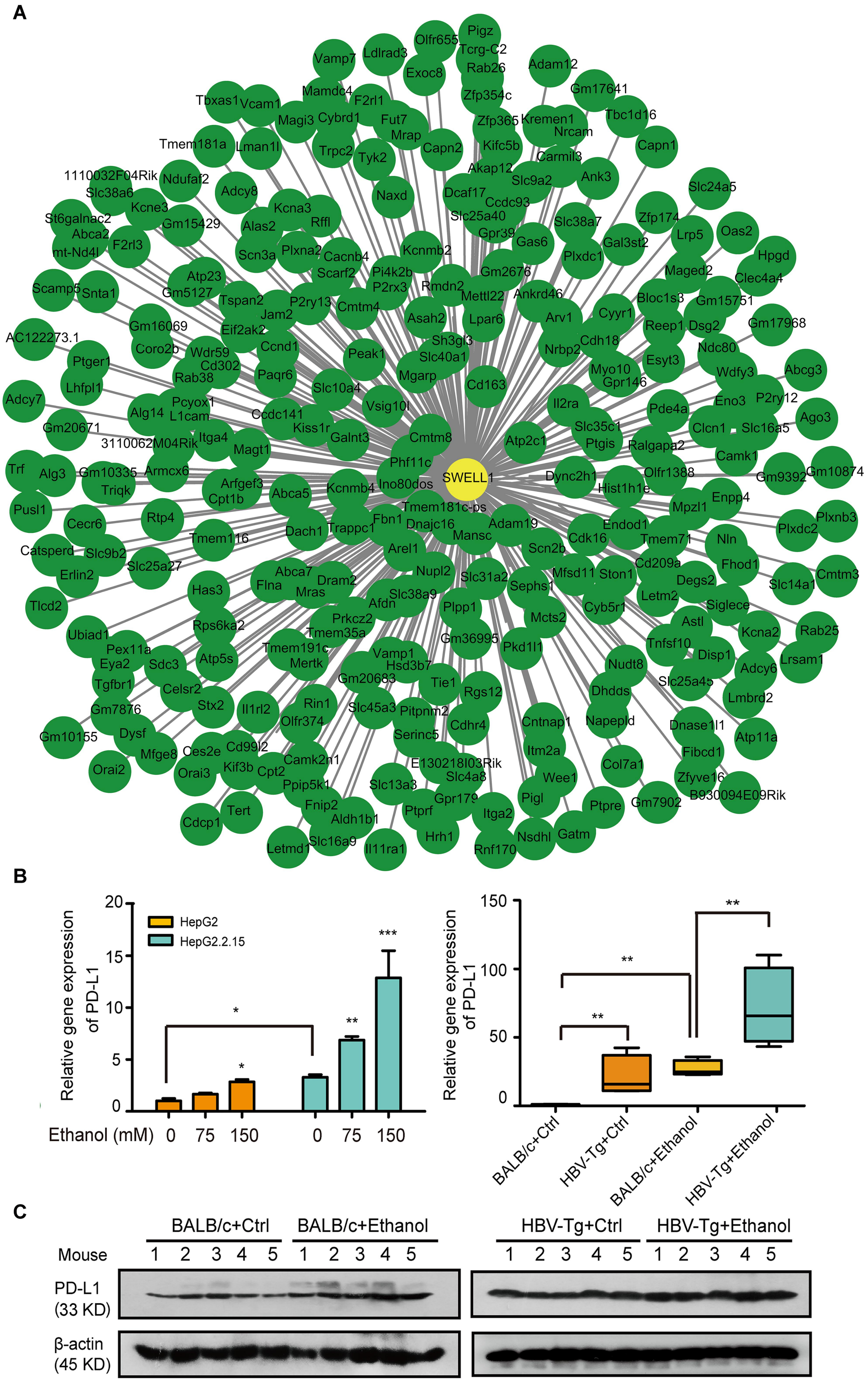

Figure S7 related to Figure 6. Profiling of Tregs induced by chronic ethanol consumption or

HBV in mice. (A) GO biological process analysis of the overlapping protein coed by SWELL1. The GO analysis was based on the DEG list. (B) Left panel: gene expression analysis of PD-L1 by RT-qPCR in HepG2 and HepG2.2.15 cells treated with dose-dependent ethanol, respectively. Right panel: gene expression analysis of PD-L1 by RT-qPCR in each groups of mice. (C) Representative Western blot analysis of PD-L1 protein levels in each groups of mice. All experiments were repeated at least three times. Error bars represent means ± SD (n = 3). *$P < 0.05$, **$P < 0.01$, ***$P < 0.001$; Student's *t*-test.

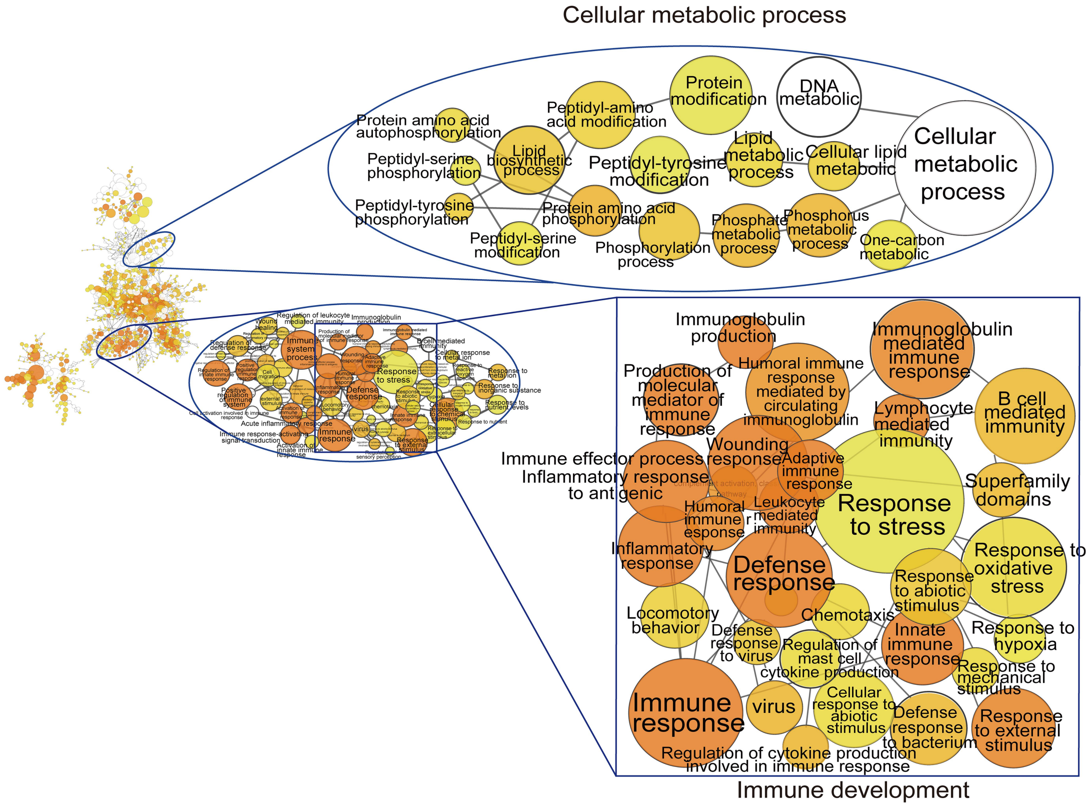

Figure S8 related to Figure 6C. GO enrichment analysis of the ethanol-induced DEGs. The up-regulated genes were analyzed for GO term enrichment by gene-set enrichment analysis. The results were visualized on a network of gene sets (nodes) connected by their similarity (edges).

Table S1. The characteristics of patients

No.	Age	Gender	Organ	HBsAg	Diagnosis
1	59	M	Liver	+	HCC
2	53	M	Liver	+	HCC
3	69	M	Liver	+	HCC
4	53	F	Liver	+	HCC
5	59	M	Liver	+	HCC
6	60	F	Liver	+	HCC
7	67	M	Liver	+	HCC
8	47	M	Liver	+	HCC
9	66	M	Liver	+	HCC
10	60	M	Liver	+	HCC
11	55	M	Liver	+	HCC
12	56	M	Liver	+	HCC
13	49	M	Liver	+	HCC
14	47	M	Liver	+	HCC
15	38	M	Liver	+	HCC
16	69	M	Liver	+	HCC
17	81	M	Liver	+	HCC
18	61	M	Liver	+	HCC
19	59	M	Liver	+	HCC
20	66	M	Liver	+	HCC
21	59	M	Liver	+	HCC
22	57	F	Liver	+	HCC
23	51	M	Liver	+	HCC
24	38	M	Liver	+	HCC
25	56	M	Liver	+	HCC
26	49	M	Liver	+	HCC
27	58	F	Liver	+	HCC
28	46	M	Liver	+	HCC
29	56	M	Liver	+	HCC
30	54	M	Liver	+	HCC

Table S2. List of primers used in this paper.

Gene	Primer	Sequence (5′-3′)
**RT-qPCR**		
Homo GAPDH	Forward	AACGGATTTGGTCGTATTG
	Reverse	GGAAGATGGTGATGGGATT
Homo SWELL1	Forward	CCAGCAGAAAGGGAACCAGGAG
	Reverse	CGGAGGGCTTGGCTTCTACTTG
HBx/pgRNA	Forward	ATGGCTGCTAGGCTGTGC
	Reverse	TTAGGCAGAGGGGAAAAAGTTG
Homo PD-L1	Forward	CCAGGATGGTTCTTAGACTCCC
	Reverse	TTTAGCACGAAGCTCTCCGAT
Homo SCD1	Forward	CCCCACCTACAAGGATAAGGA
	Reverse	CACGAGCCCATTCATAGACAT
Homo GGT5	Forward	GTCAGCCTAGTCCTGCTGG
	Reverse	GGATGGCTCGTCCAATATCCG
Homo PTGES2	Forward	GCGGCCATGTACCTCATCAG
	Reverse	AAATCAGCGAGATTCGGCTTC
Homo SCAP	Forward	GTGGACTCTGACCGCAAACAA
	Reverse	CGGGACAAAGGTGAACGAAATAC
Homo CPT1A	Forward	TCCAGTTGGCTTATCGTGGTG
	Reverse	TCCAGAGTCCGATTGATTTTTGC
Mouse GAPDH	Forward	CCTGCCAAGTATGATGACAT
	Reverse	GTTGCTGTAGCCGTATTCA
Mouse SWELL1	Forward	GGCCCCACAGGTATCAAGTA
	Reverse	GTGTGCAGAAGCACGAGGTA
Mouse PD-L1	Forward	GCTCCAAAGGACTTGTACGTG
	Reverse	TGATCTGAAGGGCAGCATTTC

Mouse SCD1	Forward	CGGTCATCCCATCGCCTGCTCT
	Reverse	GTAGGCGAGTGGCGGAACTGC
Mouse PTGES2	Forward	CCTCGACTTCCACTCCCTG
	Reverse	TGAGGGCACTAATGATGACAGAG
Mouse CPT1A	Forward	CTCCGCCTGAGCCATGAAG
	Reverse	CACCAGTGATGATGCCATTCT
Mouse SCD4	Forward	GCCCACTTGCCACAAGAGAT
	Reverse	GTAGCTGGGGTCATACAGATCA
Mouse pik3r6	Forward	AGCAATCAGGGCATGTGGAG
	Reverse	CGTCCGTCCTCGCTTTCTG
Mouse IL1B	Forward	TTCAGGCAGGCAGTATCACTC
	Reverse	GAAGGTCCACGGGAAAGACAC
Mouse Hilpda	Forward	TGCTGGGCATCATGTTGACC
	Reverse	TGACCCCTCGTGATCCAGG
**Luciferase assay**		
pGL3-SWELL1-pr	Forward	CCGCTCGAGCCCTGGCTATGATATTGCTAAGT
omoter	Reverse	CCCAAGCTTGGCGTGTGTAAGTGGGTTTC
pGL3-F1	Forward	CCGCTCGAGTGCAAGCTCCACCTCCCG
	Reverse	CCCAAGCTTGGCGTGTGTAAGTGGGTTTC
pGL3-F2	Forward	CCGCTCGAGTATCAGCCAGGGAATTTGCG
	Reverse	CCCAAGCTTGGCGTGTGTAAGTGGGTTTC
pGL3-F3	Forward	CCGCTCGAGTGCAGCAGTCCCTGTCCCTTTA
	Reverse	CCCAAGCTTGGCGTGTGTAAGTGGGTTTC
pGL3-SWELL1-pr	Forward	CCGCTCGAGCCCTGGCTATGATATTGCTAAGT
omoter-CR-wt	Reverse	CCCAAGCTTCGGGAGGTGGAGCTTGC

Table S3. List of siRNAs used in this paper.

Gene		Sequence (5′-3′)
si-SWELL1	Sense	GGUACAACCACAUCGCCUA
si-Sp1	Sense	GCCAAUAGCUACUCAACUA
si-HBx	Sense	GCAUUGUUCACCUCACCAU
si-Ctrl	Sense	UUCUCCGAACGUGUCACGU

Table S4. List of antibodies used in this paper.

Antibody	Manufacture	Catalog number
SWELL1 Rabbit Polyclonal Antibody	Abcam	ab157489
HBx Rabbit Polyclonal Antibody	Abcam	ab2741
PD-L1 Rabbit Polyclonal Antibody	Absin	Abs136046
β-actin Mouse Monoclonal Antibody	Sigma-Aldrich	A2228
IgG Rabbit Polyclonal Antibody	CST	2729S
IgG Mouse Monoclonal Antibody	CST	61656S
PerCP/Cy5.5-conjugated CD3 anti-Mouse Antibody	Biolegend	100218
PE-conjugated CD4 anti-Mouse Antibody	Biolegend	100408
FITC-conjugated CD25 anti-Mouse Antibody	Biolegend	101908
APC-conjugated CD127 anti-Mouse Antibody	Biolegend	135012
